# The *Brassica napus* fatty acid exporter FAX1-1 contributes to biological yield, seed oil content, and oil quality

**DOI:** 10.1186/s13068-021-02035-4

**Published:** 2021-09-29

**Authors:** Zhongchun Xiao, Fang Tang, Liyuan Zhang, Shengting Li, Shufeng Wang, Qiang Huo, Bo Yang, Chao Zhang, Daojie Wang, Qing Li, Lijuan Wei, Tao Guo, Cunmin Qu, Kun Lu, Yanfeng Zhang, Liang Guo, Jiana Li, Nannan Li

**Affiliations:** 1 Chongqing Engineering Research Center for Rapeseed, College of Agronomy and Biotechnology, Southwest University, Chongqing, 400715 China; 2 College of Resources and Environment, and Interdisciplinary Research Center for Agriculture Green Development in Yangtze River Basin, Southwest University, Chongqing, 400715 China; 3 Academy of Agricultural Sciences, Southwest University, Beibei, Chongqing, 400715 China; 4 College of Biology and Chemistry, Xingyi Normal University for Nationalities, Xingyi, 562400 Guizhou China; 5 Hybrid Rapeseed Research Center of Shaanxi Province, Shaanxi, China; 6 National Key Laboratory of Crop Genetic Improvement, Huazhong Agricultural University, Wuhan, 430070 China; 7 State Key Laboratory of Crop Stress Adaptation and Improvement, Key Laboratory of Plant Stress Biology, School of Life Sciences, Henan University, Kaifeng, 475004 China

**Keywords:** *Brassica napus*, Fatty acid, Oil quality, Biological yield, Seed oil production

## Abstract

**Background:**

In the oilseed crop *Brassica napus* (rapeseed), various metabolic processes influence seed oil content, oil quality, and biological yield. However, the role of plastid membrane proteins in these traits has not been explored.

**Results:**

Our genome-wide association study (GWAS) of 520 *B. napus* accessions identified the chloroplast membrane protein-localized FATTY ACID EXPORTER 1-1 (FAX1-1) as a candidate associated with biological yield. Seed transcript levels of *BnaFAX1-1* were higher in a cultivar with high seed oil content relative to a low-oil cultivar. BnaFAX1-1 was localized to the plastid envelope. When expressed in *Arabidopsis thaliana*, *BnaFAX1-1* enhanced biological yield (total plant dry matter), seed yield and seed oil content per plant. Likewise, in the field, *B. napus BnaFAX1-1* overexpression lines (*BnaFAX1-1*-OE) displayed significantly enhanced biological yield, seed yield, and seed oil content compared with the wild type. *BnaFAX1-1* overexpression also up-regulated gibberellic acid 4 (GA4) biosynthesis, which may contribute to biological yield improvement. Furthermore, oleic acid (C18:1) significantly increased in *BnaFAX1-1* overexpression seeds.

**Conclusion:**

Our results indicated that the putative fatty acid exporter *BnaFAX1-1* may simultaneously improve seed oil content, oil quality and biological yield in *B. napus*, providing new approaches for future molecular breeding*.*

**Supplementary Information:**

The online version contains supplementary material available at 10.1186/s13068-021-02035-4.

## Introduction

*Brassica napus* (rapeseed) is an important oilseed crop for edible oils, as the oil extracted from its seeds is rich in highly unsaturated fatty acids. In addition, rapeseed oil constitutes a reasonable substitute for diesel fuel as a renewable energy source due to their chemical similarities and high energy potential [9]. Increasing rapeseed oil production per hectare would thus increase edible oil and liquid biofuel production. Indeed, substantial work has been invested into improving *B. napus* seed yield and seed oil content. Similarly, increasing total biological yield of above-ground biomass contributes to increasing seed yield and seed oil production [30].

Previous studies revealed that rapeseed seed oil production may be enhanced by overexpressing enzymes or transcription factors involved in various metabolic processes. For example, the overexpression of yeast (*Saccharomyces cerevisiae*) GLYCEROL-3-PHOSPHATE DEHYDROGENASE (GPD1) under the control of a seed-specific promoter raised seed oil content [42]. The thylakoid membrane-associated lipase, PLASTID LIPASE1 (PLIP1) was reported contributing to the export of acyl groups from plastids for seed oil biosynthesis in Arabidopsis [2, 44]. Likewise, seed oil content increased in transgenic *Arabidopsis thaliana* plants overexpressing *B. napus WRINKLED1* (*WRI1*)-like [26]. The conditional expression of *B. napus* LEAFY COTYLEDON1 (*BnLEC1*) and *LEC1-LIKE* (*BnL1L*) in developing seeds enhanced seed oil content by 2–20% with no detrimental effects on major agronomic traits [40]. Finally, the overexpression of *B. napus GROWTH REGULATING FACTOR2* (*GRF2*)-like (*BnGRF2*) improved seed oil production by regulating cell number and plant photosynthesis [25].

Biological yield reflects the total accumulation of photosynthetic products in plant tissues. In plants, photosynthesis and fatty acid biosynthesis take place in chloroplasts [20]. A recent study revealed that altering carbon metabolism in the chloroplasts of transgenic tobacco (*Nicotiana tabacum*) plants with high leaf oil levels caused biological yield and oil production to increase. We speculated that photosynthetic products transported across the chloroplast membranes may be critical for biological yield. We previously conducted a genome wide association study (GWAS) of biological yield with 520 *B. napus* accessions, and identified 88 single-nucleotide polymorphisms (SNPs) that are significantly associated with this trait [30]. Few proteins have been reported that localize to the chloroplast envelope and function as transporters regulating carbon metabolism across the chloroplast membranes. In previous work, we showed that *Arabidopsis* FATTY ACID EXPORTER 1 (AtFAX1) localized to the chloroplast inner envelope and mediated fatty acid export from chloroplasts, an essential step in the biosynthesis of leaf and stem lipids [21]. Subsequently, Tian et al. [41] overexpressed *AtFAX1* under the control of a seed-specific promoter in *Arabidopsis* and demonstrated that seed-specific overexpression of *AtFAX1* could increase seed size, weight, oil and protein content. Cai et al. [4] overexpressed *AtFAX1* in *Camelina sativa*, which increased the plant biomass, seed weight, seed length, seed yield, seed oil content, and oil yield in *camelina sativa*. However, the function of *FAX1* homologous in *Brassica napus* has not been reported.

To determine the contribution of chloroplast envelope proteins to biological yield, which might increase seed oil production in *B. napus*, we identified genes encoding proteins predicted to localize to the chloroplast envelope that mapped near significant SNPs from our previous GWAS results in *B. napus*. Notably, *BnaFAX1-1*, the *B. napus* ortholog of *Arabidopsis FAX1*, mapped near a significant SNP associated with biological yield. We propose that BnaFAX1-1 is a fatty acid exporter associated with biological yield in *B. napus* based on functional annotation analysis, subcellular localization and transcript levels across various *B. napus* cultivars. Our results revealed that *BnaFAX1-1* significantly contributed to biological yield and improved seed oil production and oil quality. Furthermore, we observed that *BnaFAX1-1* may modulate gibberellic acid 4 (GA4) content, and offer a potential mechanism for the increase in biological yield. The present study provides an important solution to simultaneously improve biological yield, seed oil content, seed yield and oil quality in *B. napus* by manipulating a single gene: *BnaFAX1-1*.

## Results

### Identification of chloroplast membrane-localized proteins potentially contributing to biological yield in *B. napus*

We previously detected SNPs that are significantly associated with biological yield during a GWAS of 520 *B. napus* accessions [30]. We selected 6627 candidate genes contained within the intervals surrounding 88 significant quantitative trait loci (QTLs) associated with biological yield trait. Of those, 29 encoded proteins with potential localization to the chloroplast membrane, as determined by GO analysis (Additional file 1: Figure S1, Tables S1, S2). We further narrowed down the list of candidates to potential transporters that may be involved in the export of photosynthetic products. Notably, we identified two genes, *BnaA04g02480D* and *BnaA07g17240D* that were closely linked with the significant SNP Bn-A07-p12412116 [30] and encoded orthologous to the *Arabidopsis* membrane protein FAX1, known to mediate plastid fatty acid export.

We focused on the characterization of these two *FAX1* orthologous genes. The FAX protein family consists of seven members in *Arabidopsis*, named AtFAX1–7 [21]. To identify potential FAX orthologues in field mustard (*Brassica rapa*), wild cabbage (*Brassica oleracea*) and *B. napus*, we performed BLAST searches, using all 7 FAX protein sequences as queries, leading to the identification of 9 putative orthologs each in *B. rapa* (*BraFAX*) and *B. oleracea* (*BolFAX*), and 21 in *B. napus* (*BnaFAX*). The physicochemical characteristics (amino acid number, theoretical isoelectric point (pI) values, relative molecular weight and number of transmembrane domains) for BnaFAX proteins are listed in Additional file 1: Table S3.

**Fig. 1:**
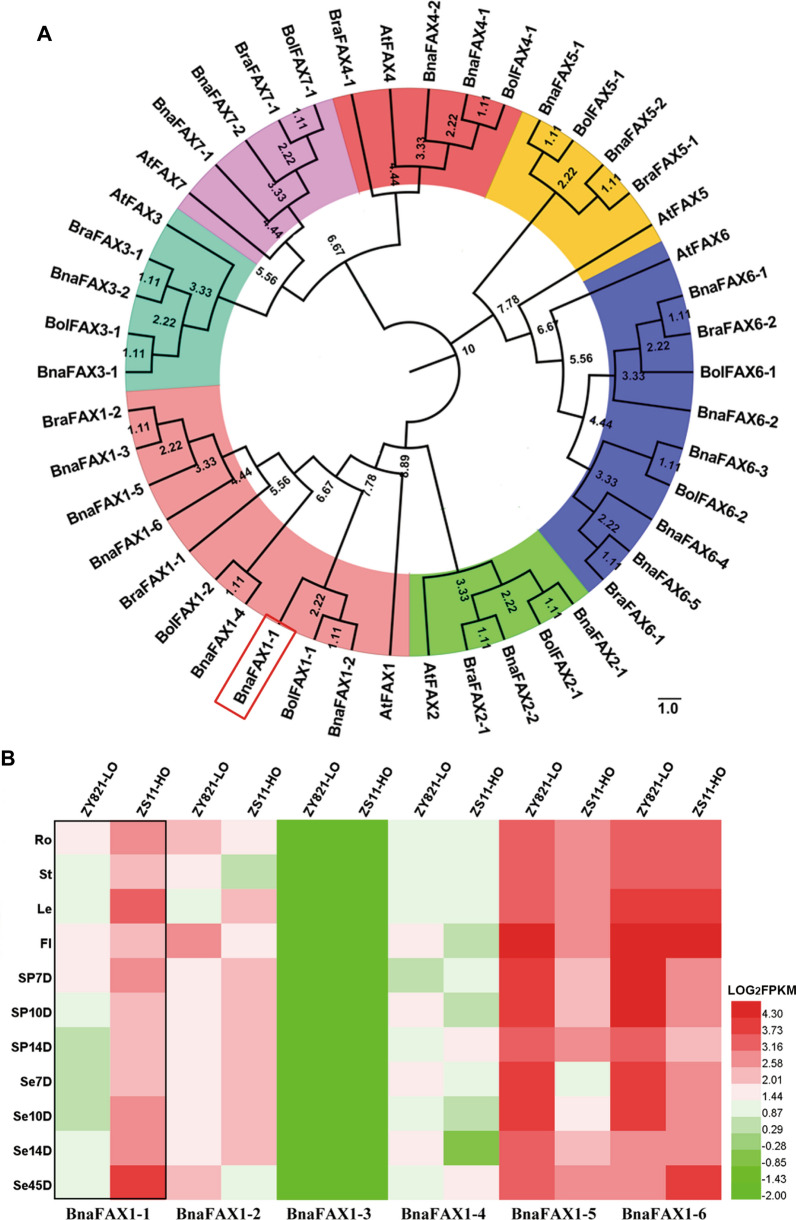
Identification and analysis of new *FAX* genes in *B. napus*. **A** Phylogenetic analysis of FAXs in *B. napus*, *B. rapa*, *B. oleracea*, and *A. thaliana*. **B** Expression analysis of *BnaFAX1* in different tissues collected from high-oil content (ZS11-HO) and low-oil content (ZY821-LO) *B. napus* genotypes. Student’s *t* test (*n* = 3–5 ± SD). Ro: Root, St: stem, Le: leaf, FL: flower, SP: siliques pericarp, SP7D: siliques pericarp after flowering 7 days, Se: seeds, Se7D: seeds after flowering 7 days

We generated an unrooted neighbor-joining phylogenetic tree based on the 46 protein sequences of FAX family members (Fig. 1A) and discovered that AtFAX1 and six putative BnaFAX1 members (BnaFAX1-1 to BnaFAX1-6) clustered into one branch. To further characterize the *B. napus* FAX family, we analyzed the chromosomal locations and gene structures of the encoding genes (Additional file 1: Figure S2A, B) and predicted the conserved motifs of BnaFAX proteins using the MEME program (Additional file 1: Figure S2C). Of the six *B. napus FAX1* members within the same branch as *AtFAX1*, *BnaFAX1-1* (*BnaA07g17240D*) and *BnaFAX1-2* (*BnaCnng07490D*) were the closest to *AtFAX1*, as evidenced by their highly similar gene structures and conserved protein motifs, suggesting that *BnaFAX1-1* and *BnaFAX1-2* may share the same functions as AtFAX1.

To determine potential effects of the six *AtFAX1*-like genes on seed oil content, we analyzed their transcript levels across various tissues in one cultivar with high seed oil contents (H, cultivar name: ZS11) and one with low seed oil content (L, cultivar name: ZY821). *BnaFAX1-1* was more highly expressed in the H cultivar relative to the L cultivar in all tissues tested (Fig. 1B). By contrast, *BnaFAX1-3* (*BnaA04g02480D*) was barely detectable in either *B. napus* cultivar. Besides, we also observed the expression levels of 6 members of *BnaFAX1* in 6 tissues of a pair of high- and low-seed oil content accessions grown in Chongqing (CQ24, CQ45) and Yunnan (YN24, YN45), among which CQ24 (seed oil content about 43%) and YN24 (seed oil content about 45%) are high-seed oil content (H-SOC) accessions, and CQ45(seed oil content about35%) and YN45 (seed oil content about 37%) are low-seed oil content (L-SOC) accessions. As shown in Additional file 1: Figure S3, the expression level of *BnaFAX1-1* in H-SOC accessions (CQ24, YN24) is higher than that of L-SOC accessions (CQ45, YN45) in the stem (St), leaf (Le), silique pericarps (30ZP) and seeds (30ZS) on the main inflorescence of 30 days after flowering and on the primary branch (30CP and 30CS) (Additional file 1: Figure S3A, C). This further confirms the conjecture that *BnaFAX1-1* may contribute to the formation of seed high oil content in *B. napus*. Furthermore, to further determine whether *BnaFAX1-1*and *BnaFAX1-2* are conducive to the formation of high biological yield (Additional file 1: Figure S1), the seedling leaves of four pairs with extremely high- (P281, P542, P125, P257-HBY) and low-biological yield accessions (P319, P276, P131, P81-LBY) were selected for qRT-PCR analysis, and biological yield dry weight per plant for each accession is shown in Additional file 1: Figure S3G. The qRT-PCR results showed that the expression levels of *BnaFAX1-1* and *BnaFAX1-2* in high-biological yield accessions were higher than those in low-biological yield accessions (Additional file 1: Figure S3E, F), which is consistent with the GWAS analysis result (Additional file 1: Figure S1). To further determine whether *BnaFAX1-1* can increase both seed oil content and biological yield, we characterized the function of *BnaFAX1-1*.

### Subcellular localization and transcript levels of BnaFAX1-1 in *B. napus*

**Fig. 2:**
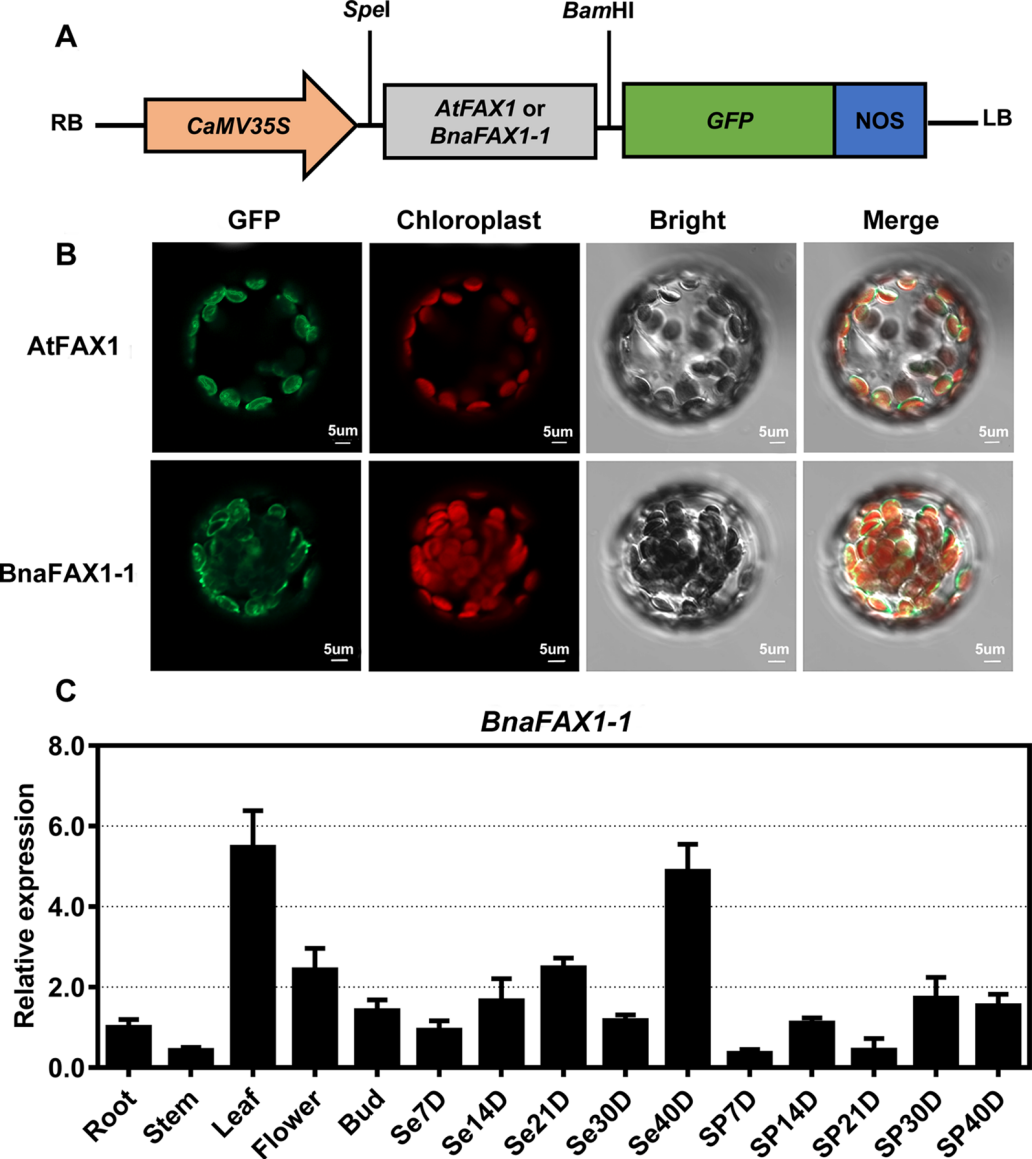
Subcellular localization of BnaFAX1-1 and expression analysis of *BnaFAX1-1* in *B. napus*. **A** Schematics of the transgene cassette bearing 35S:*BnaFAX1-1* in a modified binary vector. **B** Localization of the green fluorescent protein (GFP)-FAX1-1 protein. We transiently transfected *Arabidopsis* leaf protoplasts with constructs encoding GFP-tagged *Arabidopsis* FAX1 and *B. napus* FAX1-1. **C** Expression pattern of *BnaFAX1-1* in different tissues in *B. napus* cultivar ZS11. *P < 0.05, **P < 0.01, Student’s *t* test (*n* = 4–6; data are means ± SD)

To determine the subcellular localization of BnaFAX1-1 in plant cells, we tagged BnaFAX1-1 with green fluorescent protein (GFP) and expressed the construct under the control of the constitutive *Cauliflower mosaic virus* (CaMV) 35S promoter (Fig. 2A). We transiently transfected *Arabidopsis* protoplasts with the *BnaFAX1-1-GFP* construct, using *AtFAX1-GFP* as a marker for chloroplast envelopes. We observed a ring of fluorescence at the periphery of chloroplasts, which is consistent with a plastid envelope localization for BnFAX1-1, as seen previously with AtFAX1 (Fig. 2B).

We next measured *BnaFAX1-1* transcript levels of in seven tissues across five stages of development (roots, stems, leaves, flowers, buds, seeds, and silique pericarp after flowering at 7, 14, 21, 30 and 40 days). We observed the highest expression level for *BnaFAX1-1* in leaves and seeds after 40 days of flowering (Fig. 2C). We had previously determined that *AtFAX1* was mainly expressed in leaves, but not in seeds [21]. This result suggested that BnaFAX1-1 function may differ from that of AtFAX1, which did not contribute to seed oil accumulation in *Arabidopsis*.

### Overexpression of *BnaFAX1-1* in *Arabidopsis* and *B. napus*

**Fig. 3:**
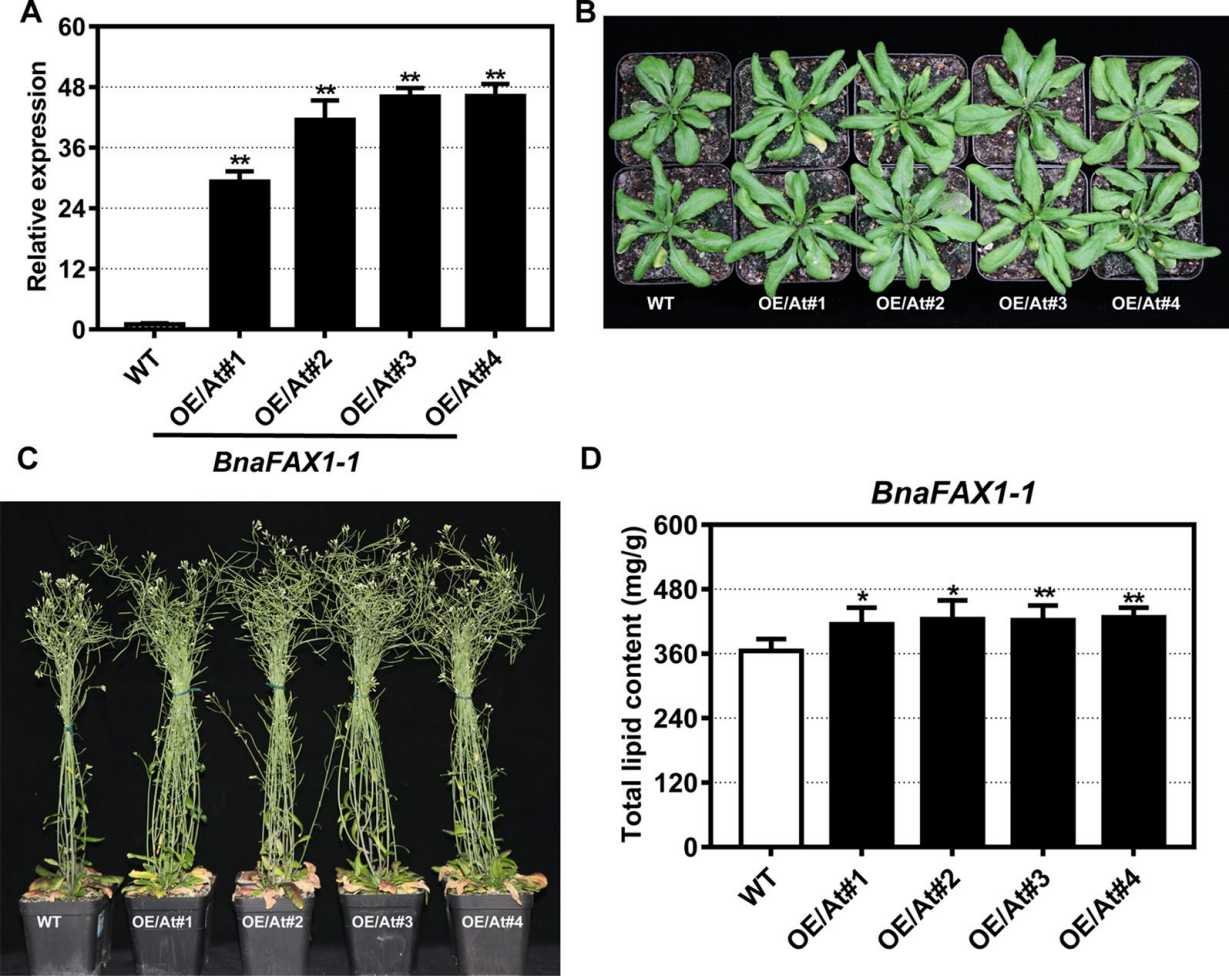
Phenotypic analysis of *Arabidopsis* lines overexpressing *BnaFAX1-1*. **A** Relative expression levels of *BnaFAX1-1* in *Arabidopsis* overexpressing lines. **B**, **C** Growth characteristics of 30-day-old (**B**) and 49-day-old plants (**C**) grown in incubators. **D** Total lipid content and fatty acid composition in mature *Arabidopsis* seeds. *P < 0.05, **P < 0.01, Student’s *t* test (*n* = 4–6 ± SD)

**Fig. 4:**
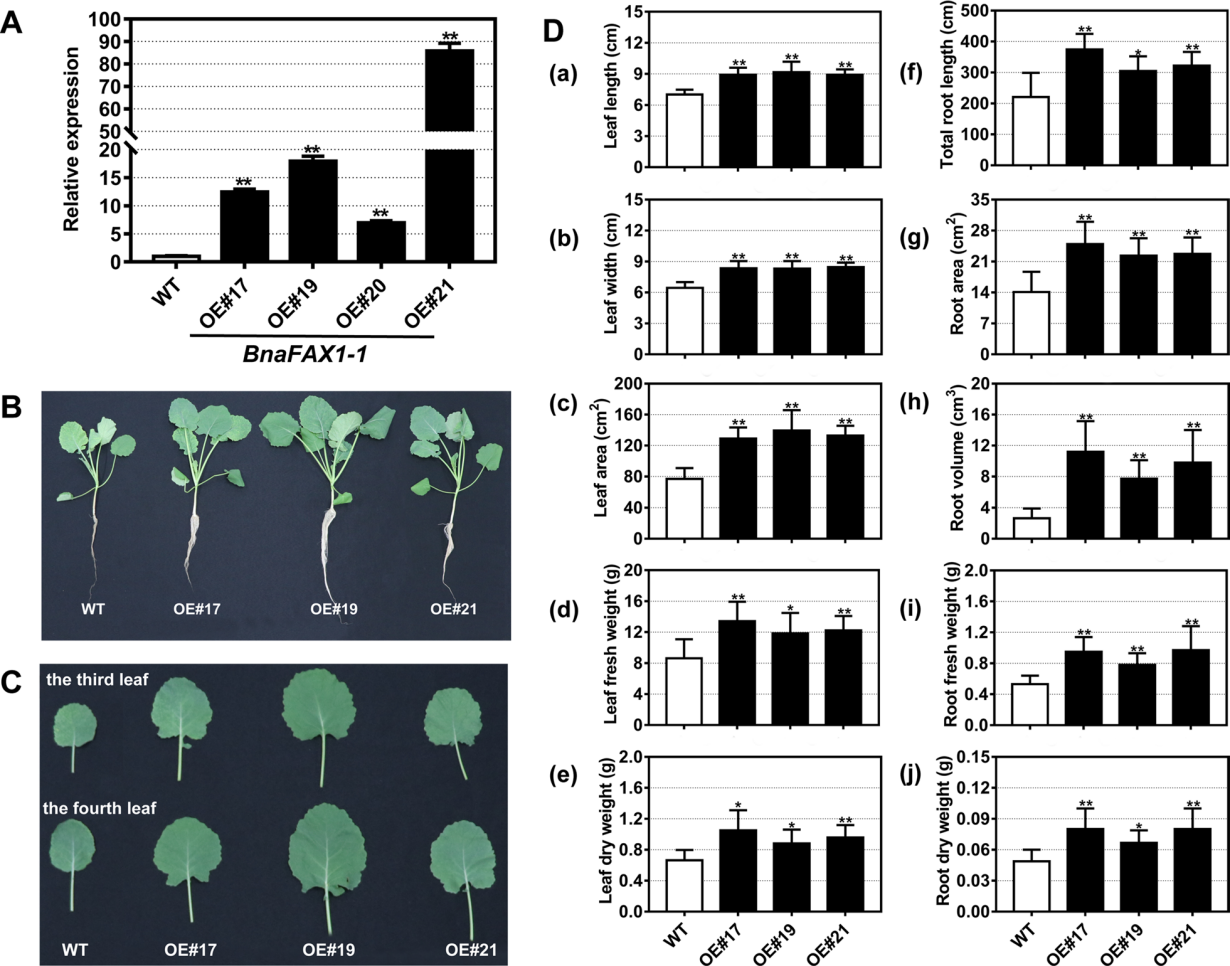
Growth and phenotypic analysis of *B. napus* lines overexpressing *BnaFAX1-1* in the plant incubator. **A** Relative expression levels of *BnaFAX1-1* in leaves from WT and transgenic lines. *BnACTIN7* was used as the internal reference. **B** Representative photographs of plants from WT and transgenic lines grown in the plant incubator for 32 days. **C** Representative photographs of the third and fourth leaves of 32-day-old plants. **D** Phenotypic analysis of WT and transgenic plants in the plant incubator. *P < 0.05, **P < 0.01, Student’s *t* test (*n* = 8 ± SD)

Next, we generated *BnaFAX1-1* overexpression constructs and transformed both *Arabidopsis* (Col-0 accession) and *B. napus* (*Westar* cultivar). We validated overexpression lines (OE) by RT-qPCR using total RNA extracted from *B. napus* and *Arabidopsis* transgenic individuals with *BnaFAX1-1*-specific primers (Additional file 1: Table S4). We selected four homozygous *BnaFAX1-1* overexpressing lines in *Arabidopsis*, named OE/At#1, OE/At#2, OE/At#3, and OE/At#4 (Fig. 3A). Similarly, we obtained four *BnaFAX1-1* overexpressing lines in *B. napus*, named OE#17, OE#19, OE#20 and OE#21 (Fig. 4A).

### Increased biological yield and seed oil production by *BnaFAX1-1* overexpression in *Arabidopsis*

Next, we analyzed the phenotype of *Arabidopsis* lines overexpressing *BnaFAX1-1* and compared their growth to wild-type (WT) plants. We noticed that all *Arabidopsis BnaFAX1-1* overexpressing lines were slightly larger and produced more biomass than WT plants (Fig. 3B, Table 1). After reaching reproductive maturity, overexpression lines were significantly bigger than the WT, with thicker inflorescence stalks and more siliques (Fig. 3C, Table 1). A detailed analysis of different tissues and organs in transgenic and WT plants grown for 7 weeks revealed that plant height, rosette fresh and dry weight, fresh and the dry weight of biological yield was significantly higher in overexpressing lines relative to WT plants (Table 1). Likewise, stem fresh weight and stem diameter in the transgenic lines were significantly increased compared to WT plants. Furthermore, we observed an increase in seed yield per plant in overexpression lines, largely due to an increase in silique number per plant (Table 1). We also determined the total lipid content of mature seeds, which indicated that overexpression lines accumulated more total lipid content relative to the WT (Fig. 3D). Collectively, these results indicated that the heterologous overexpression of *BnaFAX1-1* in *Arabidopsis* promoted plant growth and development, and led to an increase in seed oil production.

**Table 1:** Plant biomass and seed yield in *Arabidopsis* Col-0 plants and *Arabidopsis* lines overexpressing *BnaFAX1-1*

Traits	WT	OE/At#1	OE/At#2	OE/At#3	OE/At#4
Stem fresh weight (mg/cm)	12.57 ± 0.79	12.63 ± 2.03	15.31 ± 2.00**	15.9 ± 1.28**	15.86 ± 2.66**
Stem diameter (mm)	0.97 ± 0.11	1.04 ± 0.08	1.16 ± 0.99**	1.23 ± 0.14**	1.21 ± 0.08**
Plant height (cm)	32.6 ± 3.37	39.74 ± 2.21**	38.9 ± 1.13**	37.75 ± 2.5**	37.49 ± 2.37**
Rosette fresh weight (g)	1.09 ± 0.14	1.28 ± 0.17*	1.43 ± 0.3*	1.58 ± 0.36**	1.44 ± 0.32*
Rosette dry weight (g)	0.17 ± 0.02	0.18 ± 0.02	0.22 ± 0.02**	0.23 ± 0.05**	0.23 ± 0.04**
Fresh weight of BYAG (g)	2.91 ± 0.24	4.44 ± 0.48**	4.20 ± 0.45**	4.85 ± 0.57**	4.31 ± 0.43**
Dry weight of BYAG (g)	0.45 ± 0.05	0.65 ± 0.07**	0.68 ± 0.04**	0.76 ± 0.10**	0.65 ± 0.14**
Length of siliques (cm)	1.6 ± 0.09	1.57 ± 0.04	1.62 ± 0.09	1.67 ± 0.07	1.59 ± 0.1
Number of siliques per plant	349.7 ± 24.18	408.7 ± 32.1**	416.4 ± 39.05**	501.9 ± 45.95**	412.75 ± 57.54*
Number of seeds per silique	61.06 ± 6.24	64.33 ± 3.56	60.78 ± 4.93	63.33 ± 5.69	61.94 ± 6.63
Weight of per 1000 seeds (mg)	19.15 ± 0.28	18.37 ± 0.68	18.58 ± 0.11	19.12 ± 0.12	19.36 ± 0.58
Seed yield per plant (mg)	118.5 ± 34	179.8 ± 45**	174.9 ± 54*	241.7 ± 51**	172.0 ± 42*

BYAG: biological yield of above ground organs

^*^P < 0.05, **P < 0.01, Student’s *t* test (*n* = 6–10 ± SD)

### Increased biological yield, gibberellin and leaf lipid contents in *B. napus* plants overexpressing *BnaFAX1-1*

To test the effect of *BnaFAX1-1* overexpression in *B. napus* on biomass accumulation, we analyzed the growth kinetics of three independent *BnaFAX1-1* overexpression lines selected at random (OE#17, OE#19 and OE#21) (Fig. 4A). We grew all plants hydroponically in Hoagland nutrient solution for 32 days. All *BnaFAX1-1* overexpression lines were larger and produced more biomass than their non-transgenic WT control (Fig. 4B). This increase in leaf biomass was reflected in all phenotypes measured: leaf fresh/dry weight and leaf size (including leaf length, leaf width and leaf area; Fig. 4C, D). Compared to the WT, overexpression of *BnFAX1-1* also resulted in a significant increase in total root length, root area, root volume, root fresh and dry weight (Fig. 4D). Overexpression of *BnaFAX1-1* in *B. napus,* therefore, promoted plant growth.

**Fig. 5:**
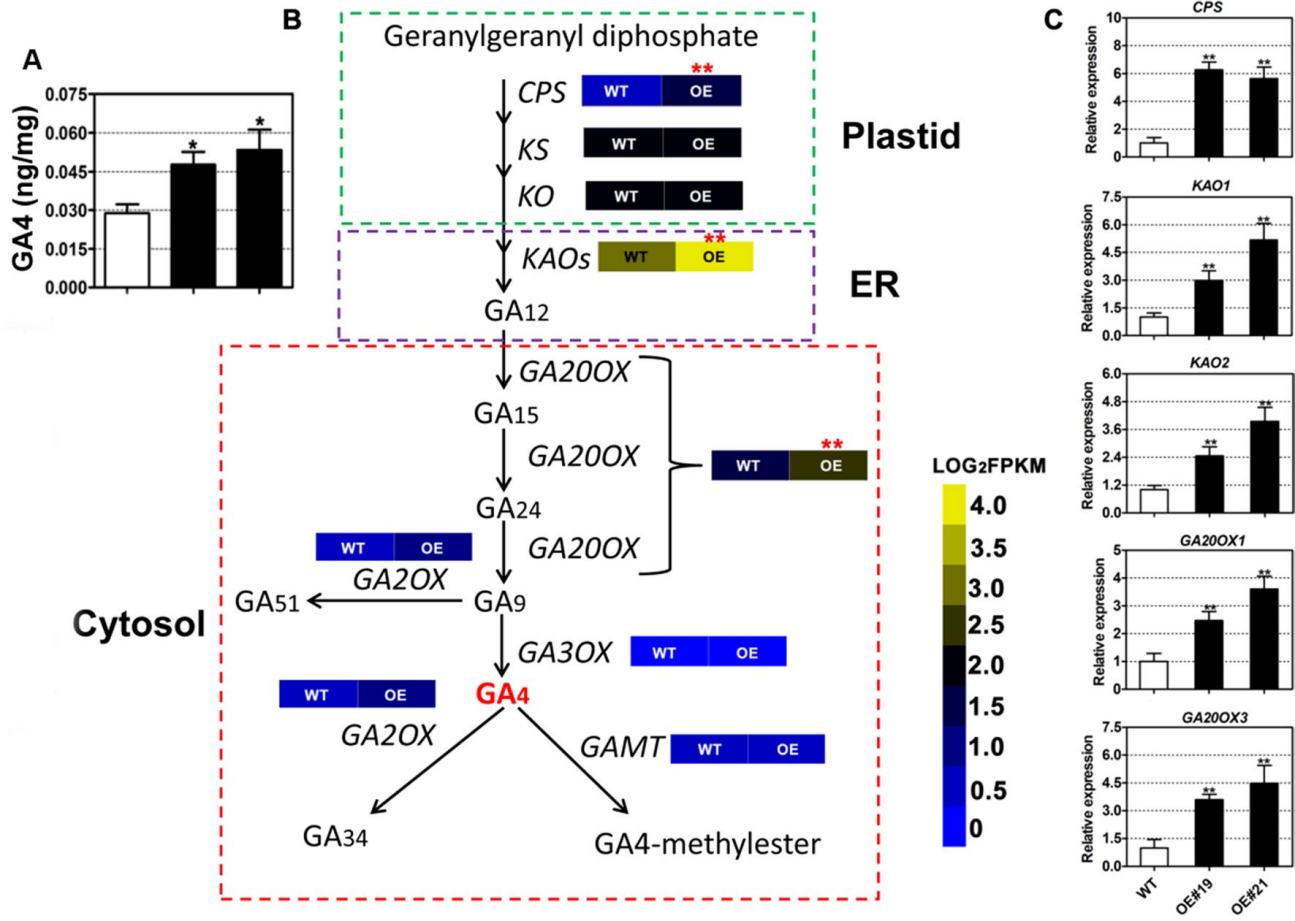
Overexpression of *BnaFAX1-1* in *B. napus* upregulates gibberellic acid biosynthesis, which may contribute to biological yield increase. **A** GA4 (gibberellic acid A4) content in leaves from 32-day-old WT and *B. napus* lines overexpressing *BnaFAX1-1*. **B** Expression patterns of GA4 biosynthetic and metabolic genes. The yellow indicates higher gene expression levels and the blue indicates lower gene expression levels. *COPALYL DIPHOSPHATE SYNTHASE*, *CPS*; *KAURENE SYNTHASE*, *KS*; *KAURENE OXIDASE*, *KO*; *KAURENOIC ACID OXIDASES*, *KAOs*; *GA 20-OXIDASE*, *GA20OX*; *GA 2-OXIDASE*, *GA2OX*; *GA 3-OXIDASE*, *GA3OX*; *GIBBERELLIC ACID CARBOXYL METHYLTRANSFERASE*, *GAMT*. **C** Confirmation of the relative expression levels of selected genes by RT-qPCR. *P < 0.05, **P < 0.01, Student’s *t* test (*n* = 4–6 ± SD)

Toward the identification of the potential mechanism linking FAX1 and *B. napus* growth and biomass improvements, we quantified phytohormone contents in the leaves of two transgenic lines (OE#19 and OE#21) and their WT using liquid chromatography followed by tandem mass spectrometry (LC–MS/MS). We observed that gibberellic acid A4 (GA4) accumulated to significantly higher levels in transgenic leaves relative to the WT (Fig. 5A). By contrast, the contents of indole-3-acetic acid (IAA), salicylic acid (SA), and jasmonic acid (JA) were similar in the overexpression lines and the WT (Additional file 1: Figure S4). GA4 is a bioactive gibberellin that plays critical roles in plant growth and development [11]. To explore the reason behind the increase in GA4 content in the transgenic lines, we performed transcriptome sequencing from leaves of the transgenic lines (OE#19 and OE#21) and WT. We discovered that the GA4 biosynthetic genes *COPALYL DIPHOSPHATE SYNTHASE* (*CPS*), KAURENOIC ACID OXIDASES (*KAOs*) and *GA20 OXIDASE* (*GA20OX*) were more highly expressed in the transgenic lines relative to the WT (Fig. 5B). We validated these results by RT-qPCR (Fig. 5C). These results indicate that overexpression of *BnaFAX1-1* led to up-regulated GA4 biosynthesis, which may in turn contribute to biological yield increase in *B. napus*.

**Fig. 6:**
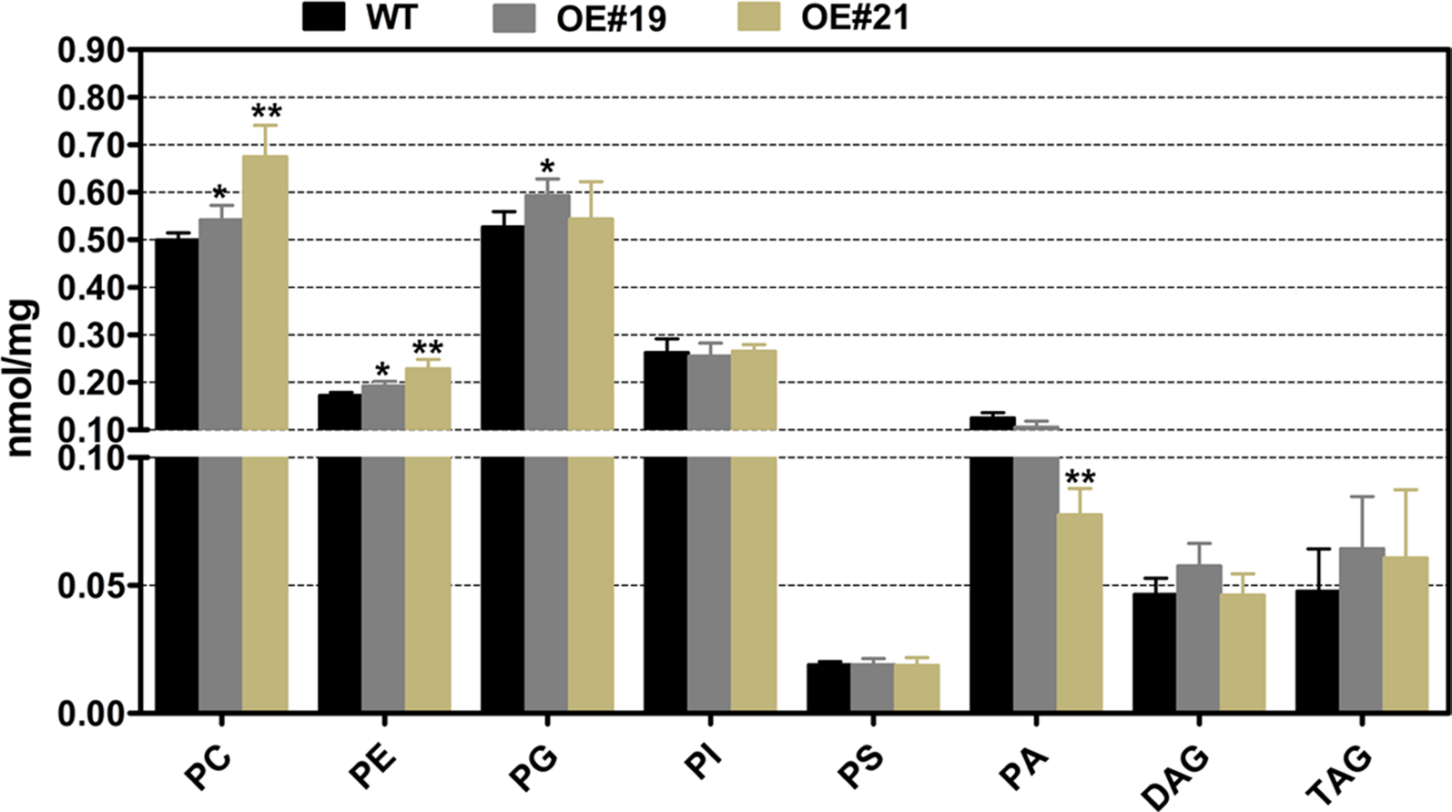
Accumulation of major polar membrane lipids species in leaves from 32-day-old WT and lines overexpressing *BnaFAX1-1.* phosphatidylcholine, PC; phosphatidylethanolamine, PE; phosphatidylglycerol, PG; phosphatidylinositol, PI; phosphatidylserine, PS;, phasphatidic acid, PA; diacylglycerol, DAG; triacylglycerol, TAG. *P < 0.05, **P < 0.01, Student’s *t* test (*n* = 4–6 ± SD)

To investigate the consequence of BnaFAX1-1 accumulation in the two selected overexpression lines above on membrane lipid contents, we analyzed lipids from 32-day-old leaves using LC–MS/MS (Fig. 6). We observed a higher lipid content for phosphatidylcholine (PC) and phosphatidylethanolamine (PE) in the leaves of OE#19 and OE#21 plants when compared to WT (Fig. 6). These results revealed that BnaFAX1-1 share the same role as AtFAX1 when ectopically expressed in leaves for the regulation of leaf lipid and biomass accumulation.

### *BnaFAX1-1* enhances biological yield and seed yield of *B. napus* plants grown in the field

**Fig. 7:**
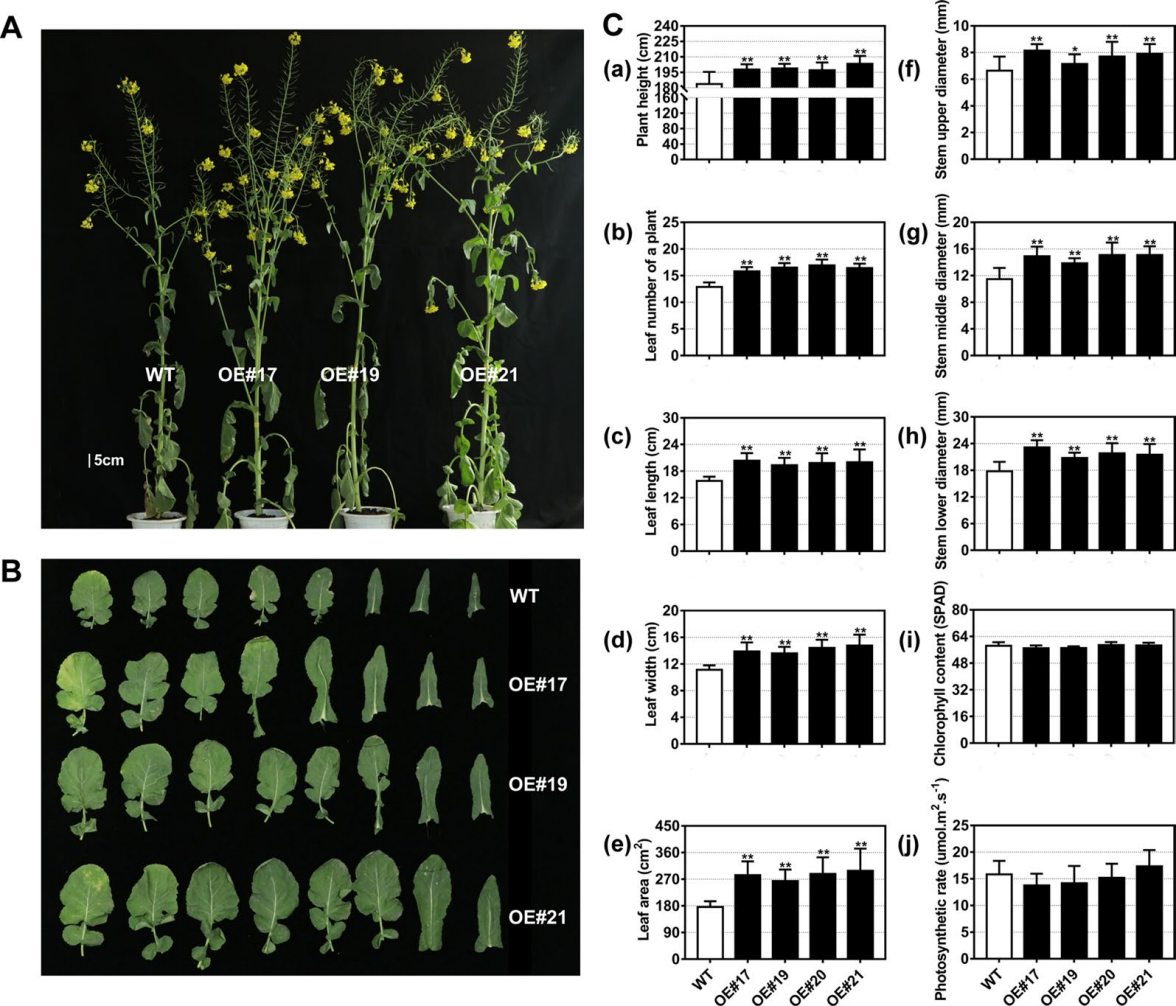
Growth of *B. napus* lines overexpressing *BnaFAX1-1* in the field. **A** Representative photographs of 175-day-old plants grown in the field. **B** Representative photographs of leaves at the same position. **C** Summary of phenotypic analysis of WT and *B. napus* lines overexpressing *BnaFAX1-1* in the field. Plant height, a; plant leaf number, b; leaf length, c; leaf width, d; leaf area, e; upper stem diameter, f; middle stem diameter, g; lower stem diameter, h; chlorophyll content i; photosynthetic rate, j. *P < 0.05, **P < 0.01, Student’s *t* test (*n* = 10 ± SD)

To determine if the phenotypes seen in *BnaFAX1-1* overexpression lines extended to the field, we sowed seeds for WT and OE lines in a randomized field plot design using four plots for each OE line and WT. We investigated growth characteristics in plants at the flowering stage (grown for 175 days). OE plants were clearly bigger and taller compared to WT, and produced more leaves on the main stem in the same growth period (Fig. 7A, C). In addition, OE plants showed larger leaves at the same position relative to WT plants, which was reflected in increased leaf length, leaf width and leaf area (Fig. 7B, C). Finally, OE lines produced thicker main stems than WT plants; only chlorophyll content and the photosynthetic rate of OE lines were similar to those of the WT (Fig. 7C).

**Fig. 8:**
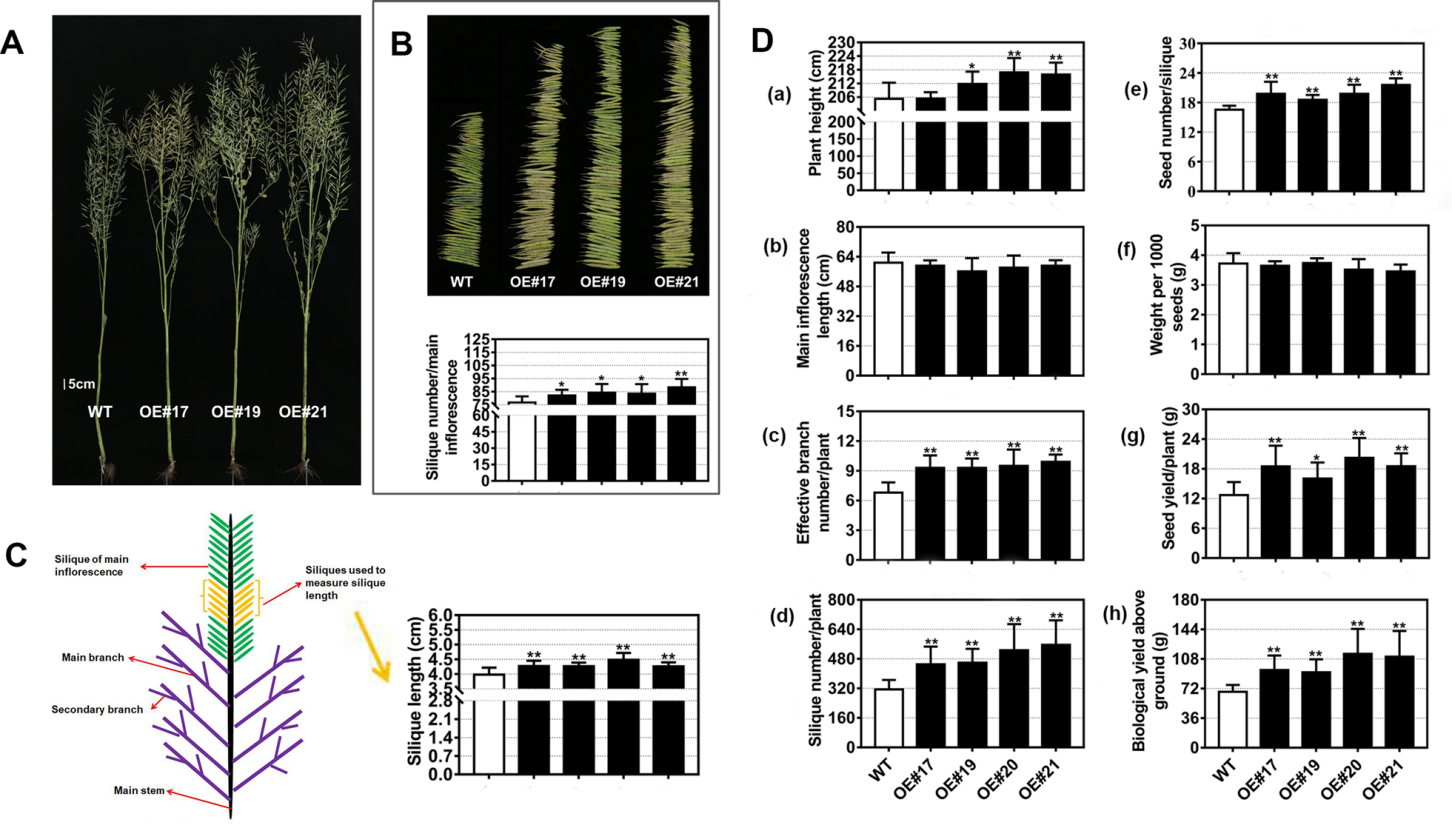
Phenotypes of *B. napus* lines overexpressing *BnaFAX1-1* after harvest in the field. **A** Representative photographs of mature plant at harvest. **B** Silique per main inflorescence from mature plants at harvest and statistic of the silique number per main inflorescence. **C** Simplified diagram of *Brassica napus* and statistic of silique length. **D** Summary of phenotypic analysis of *B. napus* WT and lines overexpressing *BnaFAX1-1* in the field. Plant height, a; main inflorescence length, b; effective branch number (seed-bearing), c; silique number per plant, d; seed number per silique, e; weight per 1000 seeds, f; seed yield per plant, g; biological yield above ground, h. *P < 0.05, **P < 0.01, Student’s *t* test (*n* = 10 ± SD)

We next harvested mature plants from the field to carry out additional measurements. The *BnaFAX1-1* OE lines were significantly taller than WT plants and bore more effective branches (i.e., branches bearing seeds) per plant (Fig. 8A, D-c). Although we observed no differences in the length of the main inflorescence between the WT and transgenic plants, the OE lines did exhibit more siliques per main inflorescence than in WT plants (Fig. 8B). Similarly, total silique number was significantly greater in all OE lines relative to WT (Fig. 8D-f), as were silique length (Fig. 8C) and number of seeds per silique. Together, these results revealed the greater seed yield per plant and biological yield in all OE plants (Fig. 8D), possibly by increasing the number of effective branches and siliques per plant. We did not observe such phenotypes when we overexpressed AtFAX1 in *Arabidopsis* in our previous work [21]. Therefore, BnaFAX1-1, unlike AtFAX1, may play a vital role in improving seed yield and biological yield in *B. napus*.

### *BnaFAX1-1* enhances *B. napus* seed oil production and improves oil quality

**Fig. 9:**
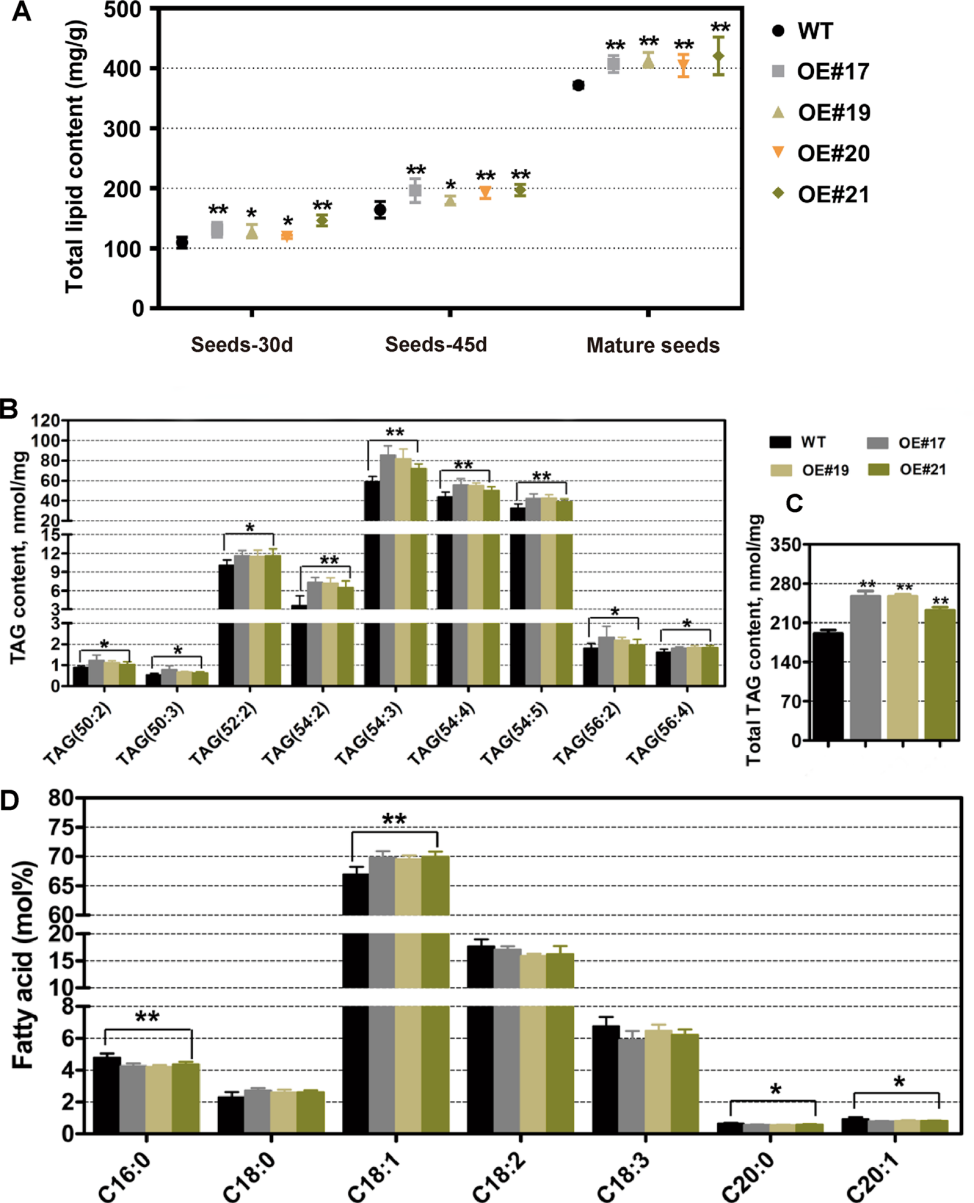
Analysis of total lipids, TAG content and fatty acid composition in *B. napus* WT and lines overexpressing *BnaFAX1-1.* **A** Total lipid content of seeds at different development stages in WT and in lines overexpressing *BnaFAX1-1*. *P < 0.05, **P < 0.01, Student’s *t* test (*n* = 4–6 ± SD). **B**, **C **Accumulation of the TAG molecular species (**B**) and total TAG content (**C**). **D** Analysis of fatty acid composition. *P < 0.05, **P < 0.01, Student’s *t* test (*n* = 6 ± SD)

We examined total lipid content in seeds from OE lines and WT at 30 d and 45 d after flowering, as well as in dry seeds following harvest. Overexpression of *BnFAX1-1* in *B. napus* resulted in an increase in total seed lipid contents at all developmental stages tested relative to WT (Fig. 9A). We also determined the range of triacylglycerol (TAG) molecular species and total TAG content in BnaFAX1-1 OE lines and WT mature dry seeds. We saw a significant rise in the content of many TAG molecular species (TAG 50:2, 50:3, 52:2, 54:2, 54:3, 54:4, 54:5, 56:2, 56:4) and total TAG content in BnaFAX1-1 OE transgenic plants compared to WT (Fig. 9B, C). In addition, an analysis of fatty acid composition of mature dry seeds grown in the field revealed that oleic acid (C18:1) was significantly increased in the transgenic lines, whereas palmitic acid (C16:0), arachidic acid (C20:0) and eicosenic cis (C20:1) were significantly reduced. Stearic acid (C18:0), linoleic acid (C18:2) and linolenic acid (C18:3) contents were similar in the OE lines and WT (Fig. 9D). These results demonstrated that overexpression of *BnaFAX1-1* effectively increased seed oil production and oleic acid content. These results, therefore, revealed that *BnaFAX1-1* may have important application value in *B. napus* molecular breeding to improve seed oil content, oil quality, seed yield and biological yield.

## Discussion

As the third largest source of plant oil after oil palm (*Elaeis guineensis*) and soybean (*Glycine max*), rapeseed (*Brassica napus*) produces approximately 15% of edible vegetable oil worldwide [13, 27, 31]. Enhancing both seed oil production and seed oil quality is a major goal in *B. napus* breeding, which can be accomplished by improving seed yield, seed oil content and biological yield, separately or in combination. The present study provides a possible and simple solution to simultaneously improve seed oil production, oil quality, seed yield, seed oil content and biological yield in *B. napus* by raising the expression levels of a single gene. We identified the potential key gene *BnaFAX1-1* through the identification of putative chloroplast-localized transporters whose encoding genes mapped closely to significant SNPs from a previous GWAS of 520 *B. napus* accessions for biological yield*.* Increasing expression of *BnaFAX1-1* improved biological yield and seed oil production, especially oleic acid content, which has not been observed in previous studies.

Chloroplasts are the main site of photosynthesis and plant fatty acid production. Recent studies revealed that altering carbon metabolism in the chloroplasts of transgenic oil crops improved biological yield and seed oil production. We hypothesized that export of photosynthates from the chloroplast may contribute to improving biological yield and seed oil production. We, therefore, focused here on genes encoding proteins predicted to locate to the chloroplast membrane, leading to a first selection of 29 candidate genes (Additional file 1: Table S2). From further analysis of these 29 genes, we identified a gene encoding a putative transporter: *BnaA07g17240D* (*BnaFAX1-1*). The *Arabidopsis* ortholog of *BnaFAX1-1* is the putative fatty acid exporter *AtFAX1*, which is crucial for biological yield. Both biological yield and seed oil production increased in 35S:*BnaFAX1-1* transgenic *B. napus* plants grown in the field relative to WT plants, highlighting the potential of this gene to improve seed oil production in *B. napus* (Figs. 4, 6, 7, 8, 9), which the positive effect of *BnaFAX1-1*-OE in *Brassica napus* is generally consistent with prior reports on *AtFAX1* gene being overexpressed individually in *Arabidopsis* [41] and *camelina sativa* [4]. A new finding of the present study is that overexpression of *BnaFAX1-1* did not affect seed weight in *Brassica napus*, while the plant biomass and seed oil yield were increased dramatically when BnaFAX1-1 was overexpressed in rapeseed.

Furthermore, we determined that the expression of GA biosynthetic genes was up-regulated, in agreement with the higher GA4 levels measured in the OE lines (Fig. 5). GA biosynthesis is derived from geranylgeranyl diphosphate (GGDP), which is synthesized in the chloroplast. GGDP is then converted to *ent*-kaurene by *ent*-COPALYL DIPHOSPHATE SYNTHASE (CPS) and *ent*-KAURENE SYNTHASE (KS). *Ent*-kaurene is then converted to *ent*-kaurenoic acid, which is catalyzed by the plastid envelope-located *ent*-kaurene oxidase (AtKO1) [16, 47]. *ent*-kaurene acid is then exported from the chloroplast to complete GA biosynthesis on the endoplasmic reticulum (ER). Fatty acid export may affect carbon metabolism in plastids and further affect *ent*-kaurene contents or export from plastids. It’s possible that BnaFAX1 may affect the carbon-hydrate types in plastids, which further affected contents of GA precursors in plastids, such as GGDP, and related enzymes contents, such as CPS. No GA precursor exporter from plastid has been reported [15]. As AtFAX1 was reported as a plastid inner-envelope localized exporter, it’s possible that BnaFAX1-1 may also localized at inner-envelope of plastids and may export fatty acids and also *ent*-kaurene etc. It would be interesting to investigate whether BnaFAX1 may be involved in GA precursor export from plastid. GA biosynthesis was reported to improve plant biological yield [11]. BnaFAX1-1 may indirectly affect GA biosynthesis, resulting in the observed increase in biological yield and seed yield, as seen in *BnaFAX1-1* overexpression lines.

Increasing seed yield and seed oil content are two major approaches to enhance seed oil production. In *B. napus*, seed yield is largely determined by three yield component traits: silique number per plant, seed number per silique, and the weight per thousand seeds [29]. In the present study, the increase in seed yield measured for *B. napus BnaFAX1-1*-OE lines was mainly due to silique number per plant and seed number per silique, as we detected no differences in the weight per thousand seeds between *BnaFAX1-1*-OE and WT plants (Fig. 8C). Seed oil content is another essential contributor to seed oil production, which constitutes the basis for *B. napus* economic importance. Overexpressing *BnaFAX1-1* in *Arabidopsis* and *B. napus* led to a significant rise in seed oil content relative to WT (Figs. 3D, 9A–C). Therefore, overexpressing *BnaFAX1-1* has great potential to increase seed oil production in *B. napus*.

In our previous study, *Arabidopsis* lines overexpressing *FAX1* showed a pronounced increase of lipids in flowers and leaves [21]. However, the *fax1* loss of function mutation had no effects on seed oil content or seed yield [21, 23]. In the green alga Chlamydomonas (*Chlamydomonas reinhardtii*), the overexpression of either *CrFAX1* or *CrFAX2* resulted in the accumulation of intracellular TAG [24]. Recently, AtFAX2 and AtFAX4 were reported to be seed-specific transporters mediating seed embryo fatty acid export for seed oil content accumulation in *Arabidopsis* [22]. These results reveal that the tissue-specificity of *FAX* expression may contribute to lipid accumulation in specific tissues. Notably, we determined that *BnaFAX1-1* is highly expressed in siliques during the seed-filling stage in a cultivar with high seed oil content, when compared to another cultivar with low seed oil content*,* indicating a correlation between BnaFAX1-1 function and the regulation of seed oil content and seed yield (Fig. 1B). The present study reveals that BnaFAX1-1 mainly mediates seed plastid fatty acid export for the accumulation of seed TAG during the seed-filling stage, leading to the measured increase in seed oil content in the overexpression lines. By contrast, AtFAX1 had no effect on seed TAG accumulation in *Arabidopsis*, as *AtFAX1* is expressed at low levels during the seed-filling stage.

Oleic acid (C18:1) is an important unsaturated fatty acid component of *B. napus* oil with high nutritional value and good thermal stability. Oil with high oleic acid content can reduce the risk of cardiovascular disease in overweight individuals [38] and effectively prevent arteriosclerosis [35]. In addition, it is highly resistant to oxidization and degradation at high temperature and enjoys a long shelf life [39]. Such oleic acid-rich oils also emit little to no smoke when heated to high temperatures and reduce cooking times [34], display high stability during frying, and imbue frying products with excellent aroma. Aside from cooking applications, oleic acid is also a raw material for biodiesel production [37]. In the present study, we determined that oleic acid (C18:1) content significantly increased in *BnaFAX1-1*-OE seeds, while another plastid-derived fatty acid palmitic acid (16:0) decreased relative to WT (Fig. 9C). In our previous study, AtFAX1 affected the content of plastid-derived fatty acids in *Arabidopsis* leaves and flowers, but not in seeds [21]. AtFAX2 and AtFAX4 mainly mediate oleic acid (C18:1) and palmitic acid (16:0), as these two fatty acids are the major free fatty acid form in plastids [22]. However, the fatty acids composition of TAG in mature seeds of *fax2*, *fax4*, *fax2fax4* and wild types shows no obvious difference, which indicates that neither *FAX2* nor *FAX4* affects the relative probability of fatty acid composition in TAG of seeds. *Arabidopsis* ATP-BINDING CASSETTE A9 (ABCA9) mediated fatty acids and acyl-CoA import into the endoplasmic reticulum for TAG accumulation. However, the fatty acid composition and contents were not affected by a loss of function in *AtABCA9* [18]. Our results suggest that BnaFAX1-1 may selectively mediate the export of specific plastid-derived fatty acids, such as oleic acid, and the potential mechanism would be the subject of future work.

In conclusion, we identified a fatty acid export protein, BnaFAX1-1, which mediates fatty acid export from plastids in developing seeds. The overexpression of *BnaFAX1-1* significantly increased seed oil content, oil quality, seed yield and biological yield in *B. napus*. *BnaFAX1-1* overexpression lines resulted in an up-regulation of GA4 biosynthesis, indicating that *BnaFAX1-1* overexpression may influence GA biosynthesis, leading to increased biological yield and seed oil production. Furthermore, BnaFAX1-1 contributed to the accumulation of oleic acid, an unsaturated fatty acid of high economic value, in seeds, thus improving oil quality. The present study provides an important solution to simultaneously improve rapeseed seed oil production, seed oil content, seed yield, biological yield and seed oil quality by modulating the expression of *BnaFAX1-1*. We propose that *BnaFAX1-1* should be a potential target for *B. napus* molecular breeding in the future.

## Experimental procedures

### Phylogenetic analyses, protein properties and sequence analyses of the FAX family

We downloaded the sequences of FAX proteins from *Arabidopsis* (*Arabidopsis thaliana*), field mustard (*Brassica rapa*), wild cabbage (*Brassica oleracea*), and *B. napus* to generate on-multiple protein sequence alignments using the integrated MUSCLE program in MEGA7.0 [19]. We also generated a phylogenetic tree of the FAX family with MEGA7.0 software using the Neighbor-Joining (NJ) method and bootstrap analysis (1000 replications). We visualized the phylogenetic trees in FigTree v1.4.2 [1]. We deduced the physical properties of BnaFAX proteins (molecular weight [MW] and isoelectric point [pI]) using the online proteomics database ExPASy (http://web.expasy.org/peptide_mass/) [12]. We predicted transmembrane domains using the online tool TMHMM Server v. 2.0 (http://www.cbs.dtu.dk/services/TMHMM/) [6]. We determined the chromosomal location of all *BnaFAX* genes by performing Basic Local Alignment Search Tool for Nucleotides (BLASTN) searches of their sequences against the 19 *B. napus* chromosomes, followed by visualization with the MapChart Software [43]. We identified exon/intron boundaries for the *BnaFAX* genes at the Gene Structure Display Server (GSDS 2.0, http://gsds.cbi.pku.edu.cn/index.php) [14] and conserved protein motifs with the MEME suite of programs (http://meme-suite.org/) [3], respectively. We then annotated all identified conserved motifs with InterProScan (http://www.ebi.ac.uk/Tools/pfa/iprscan/) according to MAST data from the MEME program [17].

### Vector constructs and plant transformation

We PCR-amplified the complete coding sequence (CDS) of the *BnaFAX1-1* gene from *B. napus* cDNA using the primer pair BnaFAX1-1 FP(*Xba*I) + BnaFAX1-1 RP(*Sac*I) (primers used in this study are listed in Additional file 1: Table S4). We then subjected the PCR product to restriction digest with *Xba*I and *Sac*I and ligated the purified digested product into the pCAMBIA2301M vector (a modified version of pCAMBIA2301 available in our laboratory) to generate pCAMBIA2301-35S:*BnaFAX1-1*. We introduced the pCAMBIA2301-*35S:BnaFAX1-1* construct into Agrobacterium (*Agrobacterium tumefaciens*) strain GV3101 for transformation of *Arabidopsis* Col-0 accession by the floral dip method [5]. We selected positive transgenic by germinating seeds on Murashige and Skoog (MS) medium supplemented with 10 mg/L Basta. We also transformed *B. napus* (*Westar* cultivar) hypocotyls with Agrobacterium bearing the pCAMBIA2301-*35S:BnaFAX1-1* construct based on a protocol described previously [32]. We selected transgenic calli for growth on MS medium supplemented with Basta, and identified transgenic plants by PCR using *BnaFAX1-1*-specific primers (F35S3ND + BnaFAX1-1 RP (SacI), and BnaFAX1-1 FP (XbaI) + NOS5ND) (Additional file 1: Table S4). We then transferred positive 35S:*BnaFAX1-1* transgenic plants to soil for seed setting and phenotyping.

### Subcellular localization of *BnaFAX1-1-*GFP fusion protein

We PCR-amplified the *Arabidopsis FAX1* CDS from *Arabidopsis* Col-0 accession cDNA using the primer pair AtFAX1 FP (SpeI) + AtFAX1 RP (BamHI) (Additional file 1: Table S4). Similarly, we amplified the *BnaFAX1-1* CDS by PCR from *B. napus* cDNA using the primer pair BnaFAX1-1 FP(*Spe*I) + BnaFAX1-1 RP (BamHI) (Additional file 1: Table S4). We subjected both PCR products to restriction digest with SpeI and BamHI before ligation into the pAN580 vector to generate pAN580-35S:*AtFAX1*-GFP and pAN580-35S:*BnaFAX1-1*-GFP (Fig. 2A) [8], which were used to transiently transfect *Arabidopsis* protoplasts. We performed the transformation and analysis of *Arabidopsis* mesophyll protoplasts as described previously [10]. GFP fluorescence was detected at 672 to 750 nm and chlorophyll autofluorescence was monitored at 503 to 542 nm by laser scanning confocal microscopy (Carl Zeiss, LSM800). We used 35S:*AtFAX1*-GFP as a positive control for the chloroplast inner envelope.

### Plant growth conditions and trait measurements

For *Arabidopsis*, we surface-sterilized seeds from Col-0 and homozygous lines overexpressing *BnFAX1-1* (*BnaFAX1-1-OE*) and sowed them on half-strength MS medium agar plates supplemented with 1% sucrose. We incubated plates in the dark at 4 °C for 2 days before releasing them in a plant incubator set to a light/dark photoperiod of 16 h light/8 h dark. After 7 days, we transferred seedlings to soil and allowed them to grow in the incubator under the same conditions. After 7 weeks, we measured a number of phenotypes on all plants: stem fresh weight (mg/cm; 1 cm from the bottom of 2nd internode of the primary inflorescence stem), stem diameter (mm; from bottom part of 2nd internode of the primary inflorescence stem), silique length, plant height, rosette fresh/dry weight, and fresh/dry weight of biological yield above ground per plant. We also collected the number of siliques per plant, the number of seeds per silique, the weight of 1000 seeds and the seed yield per plant after seeds maturation.

For *B. napus* growth in hydroponics, we germinated seeds for *B. napus* WT (*Westar* cultivar) and BnaFAX1-1-OE lines in glassware covered with three layers of wet filter paper. We transferred 8-day-old seedlings to quarter-strength Hoagland nutrient solution in a plant incubator under a temperature cycle of 26 °C (day)/22 °C (night) and a photoperiod of 16 h light/8 h dark. We replaced the nutrient solution with half-strength Hoagland nutrient solution after 5 days, which we replaced 5 days later for full-strength Hoagland nutrient solution. We then refreshed the nutrient solution with full-strength Hoagland nutrient solution every 5 days until harvesting. After 32 days, we measured total root length, root area, root volume, root fresh/dry weight, leaf length, leaf width, leaf area, and leaf fresh/dry weight.

For field experimental work, we sowed *B. napus* WT (*Westar* cultivar) and BnaFAX1-1-OE lines in soil in Beibei, Chongqing (29°45′N, 106°22′E, 238.57 m). Plants grown in field were arranged in a randomized field plot design with three replicates per genotype. Each plot contained four rows: 10 plants per row, 30 cm between rows, and 20 cm between plants within each row. After 175 days, we measured plant height, the number of leaves on the main stem for each plant, leaf length, leaf width, leaf area, the diameter at the same position on the main stem, chlorophyll content and photosynthetic rate for all plants. After seeds maturation, we scored plant height, effective branch number per plant (i.e., branches bearing seeds), length of the main inflorescence, silique number per main inflorescence, silique number per plant, silique length, seeds per silique, weight per 1000 seeds, overall seed yield per plant, and dry weight of biological yield above ground. We harvested 8–15 plants from each plot, averaged the measurements in each plot and used the resulting data for ANOVA.

### Plant phytohormone extraction

We harvested 50–75 mg of leaf tissue from 35-day-old *B. napus* WT (*Westar* cultivar) and BnaFAX1-1-OE lines grown in incubator and extracted phytohormones using previously published methods [36]. Briefly, we mixed samples in 500–750 μL 2-propanol/water/concentrated hydrochloric acid (2: 1: 0.002 v/v/v) for extraction and it was shaken at 100 rpm for 30 min at 4 °C. We then added 1 mL dichloromethane, and it also was shaken at 100 rpm for 30 min at 4 °C, then centrifuged the mixture and collected the supernatant. We repeated this extraction procedure three times, combined all supernatants and dried them under nitrogen flow. Finally, we added 200 µL of methanol/0.1% formic acid aqueous solution (1: 1 v/v) to resuspend the pellet, filtered it through an organic filter and placed each sample into the injection tube. We analyzed phytohormones by liquid chromatography followed by tandem mass spectrometry (LC–MS/SM) (QTRAP 6500+) using the MRM approach described by Pan et al. [36] Briefly, LC uses a binary solvent system. The mobile phase is methanol and 0.05% formic acid. We selected an Eclipse plus C18 (5 μm, 2.1 * 150 mm) chromatographic column. The flow rate was controlled at 300 µL/min and the column temperature was 30 °C, 10 µL per injection. We used a gradient elution, with the initial gradient of methanol of 10%, held for 2 min, and gradually increased to 10 min and maintained at 90% for 5 min. At 15.1 min, we reduced methanol to the initial gradient and held for 7 min. We added external phytohormone standards for gibberellic acid, abscisic acid, indole-3-acetic acid, salicylic acid and jasmonic acid to calculate the level of each phytohormone in the samples.

### Lipid analyses

We extracted lipids from 32-day-old leaves and analyzed lipid content by LC–MS/MS using the method reported previously [31]. We determined the levels and molecular species for phosphatidylcholine (PC), phosphatidylethanolamine (PE), phosphatidylglycerol (PG), phosphatidylinositol (PI), phosphatidylserine (PS), phosphatidic acid (PA), diacylglycerol (DAG) and triacylglycerol (TAG) as previously described [46]. For each experiment, we sampled six plants in six technical replicates; we used 50–100 mg fresh leaf tissue to extract lipids.

We quantified fatty acid composition using a gas chromatograph coupled with a flame ionization detector (GC-FID). We immersed 3–5 mg dry seed samples in 1.5 mL methanol containing 1.5% H_2_SO_4_ and 0.01% BHT. We then added 25 µL heptadecanoic acid triglyceride (C17: 0 TAG) with 5.4 µmol/L in tubes as internal standard. We incubated the tubes in a water bath at 90 °C for 1 h before allowing them to cool to room temperature. We then added 1 mL H_2_O and 1 mL chromatographic hexane, mixed well, and centrifuged the samples at 1000 rpm for 10 min. Subsequently, we transferred 0.8 mL of the upper phase to a new glass tube and dried it under nitrogen flow. Finally, we added 0.4 mL hexane to dissolve fatty acid methyl esters, and injected 1 µL of the ester solution into the GC with the detector temperature set to 280 °C, oven temperature to 170 °C for 2 min, and then increased by 3 °C/min up to 210 °C.

We determined total lipid content in seeds using a previously described method [33]. Briefly, we immersed 50 mg dry seeds in 1 mL methanol (chromatography grade) and 2 mL 2% (m/v) NaOH in glass tubes, after which we mashed the seeds with glass rods and vortexed for 10 min on a vortex shaker. Next, we placed the glass tubes containing the seed mixture in a 60 °C water bath for 1 h before allowing the tubes to cool to room temperature. We then added 2 mL chloroform (chromatography grade) and vortexed again for 10 min. After centrifugation of the mixture at 5000*g* for 5 min at room temperature, we transferred the lower phase (chloroform) to a dry weighed glass tube, added 1 mL hexane (chromatography grade) to the remaining upper layer and vortexed again for 10 min. After centrifugation, we collected the upper phase (hexane) and added it to the transferred chloroform solution. Finally, the mixture was dried under nitrogen flow and the content of total lipid was calculated. Five biological replicates were performed for each line.

### RNA isolation and quantitative RT-PCR analysis

We extracted total RNA from various tissues of *B. napus* cultivar Zhongshuang11 (ZS11) using the EZ-10 DNAaway RNA Mini-prep Kit (Sangon Biotech (Shanghai), Co., Ltd). We then synthesized first-strand cDNAs from 1 µg total RNA using the PrimeScript™ RT reagent Kit with gDNA Eraser (Perfect Real Time) (TaKaRa Biotechnology, Dalian, China) according to the manufacturer’s instructions. We performed real-time quantitative PCR analysis using SYBR Premix Ex Taq II (Perfect Real Time) (TaKaRa, Dalian, China) in a CFX96 real-time PCR system (Bio-Rad, USA) according to previous methods [28]. Gene-specific primers were designed using Vector NTI software, with the *BnACTIN7* gene as internal reference gene (Additional file 1: Table S4) [7]. We performed RT-qPCR on three independent biological replicates, each consisting of three technical replicates. We determined *BnaFAX1-1* transcript levels in WT plants and transgenic lines by RT-qPCR as described above. In *Arabidopsis*, we used *ACTIN2* as an internal control (Additional file 1: Table S4) [45].

### Statistical analysis

All data were analyzed for statistical significance using SPSS 19.0 and GraphPad Prism 7. Analysis of variance was performed on data sets, and the values are presented as means ± SD. We used Duncan’s test or Student’s *t* test to analyze the statistical significance between WT and transgenic lines (*P < 0.05; **P < 0.01).

## Supplementary Information

**Additional file 1: Figure S1.** Identification of new *FAX* genes in *B. napus*. We selected genes encoding chloroplast membrane proteins based on significant single-nucleotide polymorphisms (SNPs) associated with biological yield in rapeseed (13/14CQ-BY: 2013/2014Chongqing-biological yield). *BnaFAX1-1* (*BnaA07g17240D*) was closely linked to the significant SNP Bn-A07-p12412116 for biological yield, according to our previous work [30]. **Figure S2.** Location, structure and conserved motif analysis of *FAXs* in *B. napus*. (A) The chromosomal distributions of the *BnaFAX* genes. (B) Gene structures of *BnaFAXs* and (C) conserved motifs analysis of BnaFAXs. **Figure S3.** Expression levels of six members of *BnaFAX1* in six tissues of a pair of high- and low-seed oil content accessions grown in Chongqing (CQ24, CQ45) (A, B) and Yunnan (YN24, YN45) (C, D), and the expression levels of Bna*FAX1-1* (E), *BnaFAX1-2* (F) in seedling leaves (120 days in field) of four pair with high- (HBY) and low-biological yield accessions (LBY). CQ24, YN24 represent high-seed oil content accessions (HO); CQ45, YN45 represent low-seed oil content accessions (LO). St, Stem; Le, Leaf; 30ZP, silique pericarps on the main inflorescence of 30 days after flowering; 30ZS, seeds on the main inflorescence of 30 days after flowering; 30CP, silique pericarps on the primary branch of 30 days after flowering; 30CS, seeds on the primary branch of 30 days after flowering. The expression levels of *BnaFAX1-1* (E), *BnaFAX1-2* (F) in seedling leaves (120 days in field) of four pair with high- (P281, P542, P125, P257-HBY) and low-biological yield accessions (P319, P276, P131, P81-LBY). The biological yield dry weight per plant for each accession (G). **Figure S4.** Phytohormone contents in leaves from 32-day-old WT and *B. napus* lines overexpressing *BnaFAX1-1*. ABA, abscisic acid; IAA, indole-3-acetic acid; SA, salicylic acid; JA, jasmonic acid. **Table S1.** Summary of significant associated SNPs and candidate genes for biological yield. **Table S2.** Chloroplast membrane proteins in candidate intervals associated with biological yield. **Table S3.** Identification of *BnaFAX* gene family members. **Table S4.** List of primer sequences used in this study.

## Data Availability

All data generated or analysed during this study are included in this published article and its supplementary information files.
