# Supplementary material for: The Brassica napus fatty acid exporter FAX1-1 contributes to biological yield, seed oil content, and oil quality
Source: Biotechnol Biofuels. 2021 Sep 29;14:190. doi: 10.1186/s13068-021-02035-4 (PMC8482660; doi:10.1186/s13068-021-02035-4)
Supplement: Supplementary file 1 — Additional file 1: Figure S1. Identification of new FAX genes in B. napus. We selected genes encoding chloroplast membrane proteins based on significant single-nucleotide polymorphisms (SNPs) associated with biological yield in rapeseed (13/14CQ-BY: 2013/2014Chongqing-biological yield). BnaFAX1-1 (BnaA07g17240D) was closely linked to the significant SNP Bn-A07-p12412116 for biological yield, according to our previous work [30]. Figure S2. Location, structure and conserved motif analysis of FAXs in B. napus. (A) The chromosomal distributions of the BnaFAX genes. (B) Gene structures of BnaFAXs and (C) conserved motifs analysis of BnaFAXs. Figure S3. Expression levels of six members of BnaFAX1 in six tissues of a pair of high- and low-seed oil content accessions grown in Chongqing (CQ24, CQ45) (A, B) and Yunnan (YN24, YN45) (C, D), and the expression levels of BnaFAX1-1 (E), BnaFAX1-2 (F) in seedling leaves (120 days in field) of four pair with high- (HBY) and low-biological yield accessions (LBY). CQ24, YN24 represent high-seed oil content accessions (HO); CQ45, YN45 represent low-seed oil content accessions (LO). St, Stem; Le, Leaf; 30ZP, silique pericarps on the main inflorescence of 30 days after flowering; 30ZS, seeds on the main inflorescence of 30 days after flowering; 30CP, silique pericarps on the primary branch of 30 days after flowering; 30CS, seeds on the primary branch of 30 days after flowering. The expression levels of BnaFAX1-1 (E), BnaFAX1-2 (F) in seedling leaves (120 days in field) of four pair with high- (P281, P542, P125, P257-HBY) and low-biological yield accessions (P319, P276, P131, P81-LBY). The biological yield dry weight per plant for each accession (G). Figure S4. Phytohormone contents in leaves from 32-day-old WT and B. napus lines overexpressing BnaFAX1-1. ABA, abscisic acid; IAA, indole-3-acetic acid; SA, salicylic acid; JA, jasmonic acid. Table S1. Summary of significant associated SNPs and candidate genes for biological yield. Table S2. [file 13068_2021_2035_MOESM1_ESM.docx]

**Supplementary files of**

**A novel fatty acid exporter BnaFAX1-1 contributes to biological yield and seed oil production of *Brassica napus* by Xiao et al.**

**Supplementary figures: 4**

**Supplementary tables: 4**


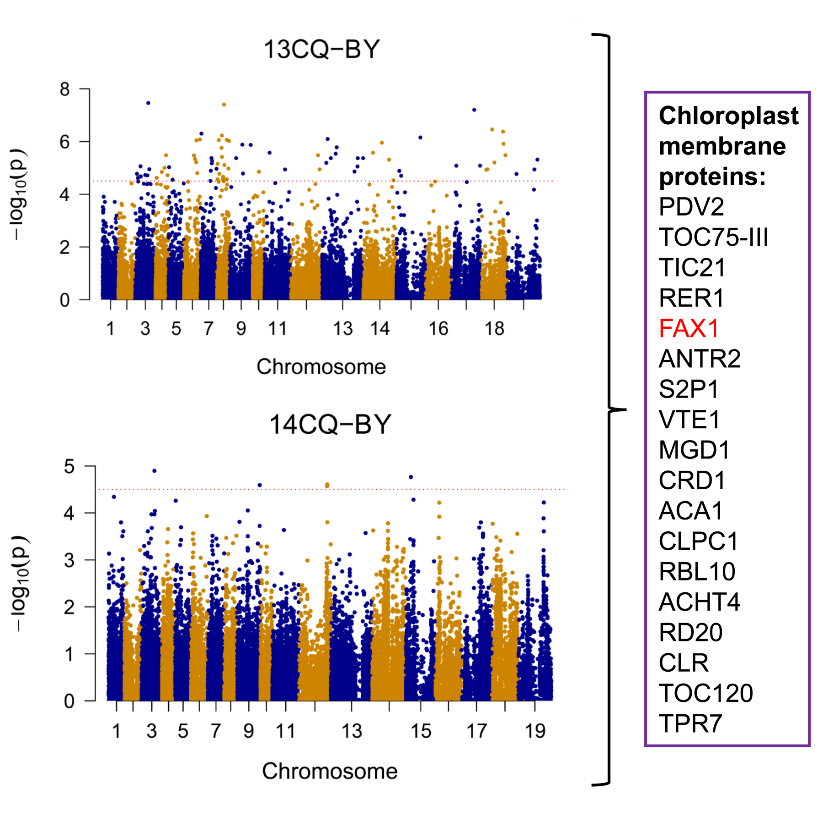


**Figure S1. Identification of new *FAX* genes in** ***B. napus***

We selected genes encoding chloroplast membrane proteins based on significant single-nucleotide polymorphisms (SNPs) associated with biological yield in rapeseed (13/14CQ-BY: 2013/2014Chongqing-biological yield). *BnaFAX1-1* (*BnaA07g17240D*) was closely linked to the significant SNP Bn-A07-p12412116 for biological yield, according to our previous work (Lu et al., 2016).


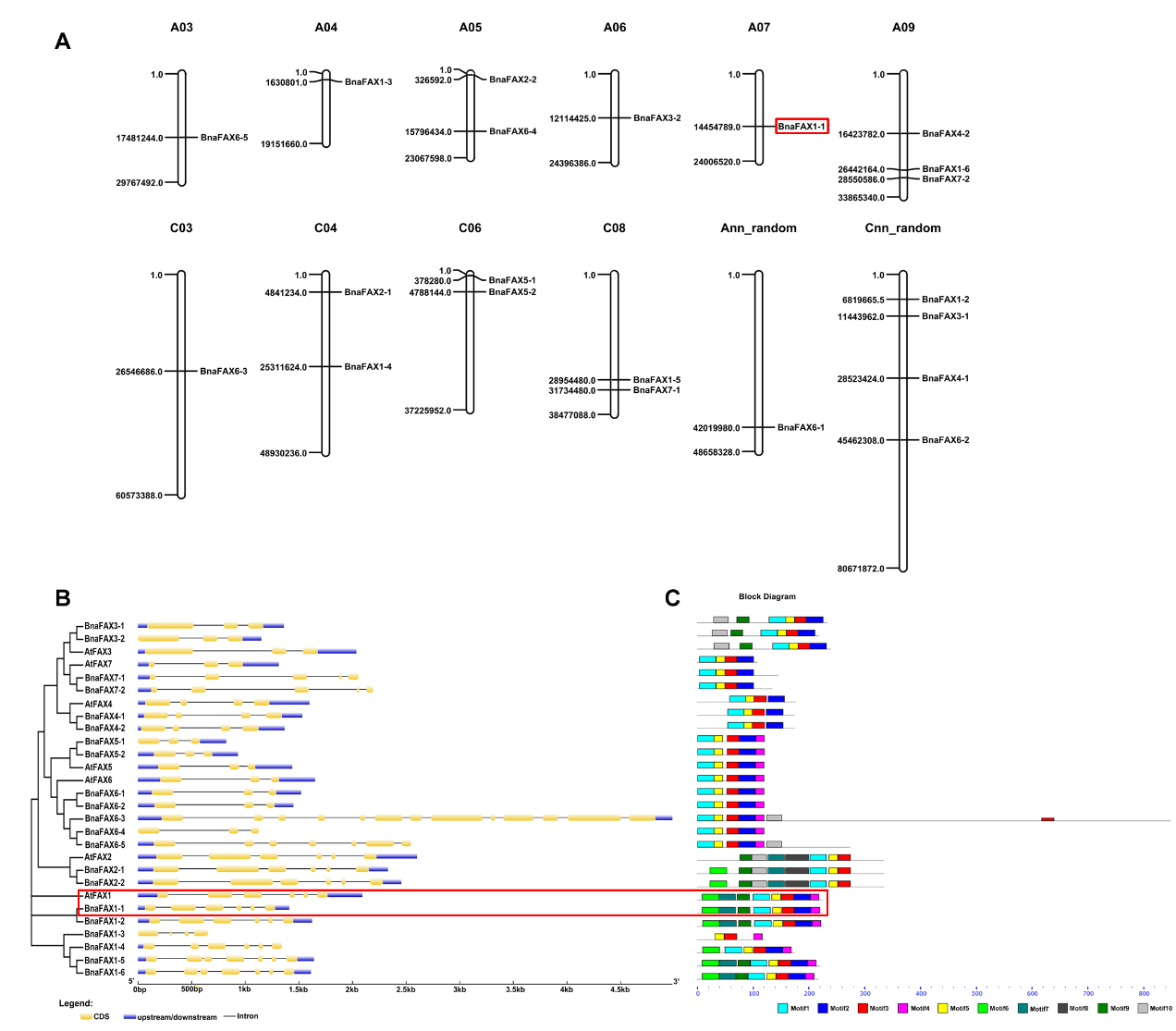


**Figure S2.** Location, structure and conserved motif analysis of *FAXs* in *B. napus*. (A) The chromosomal distributions of the *BnaFAX* genes. (B) Gene structures of *BnaFAXs* and (C) conserved motifs analysis of BnaFAXs.


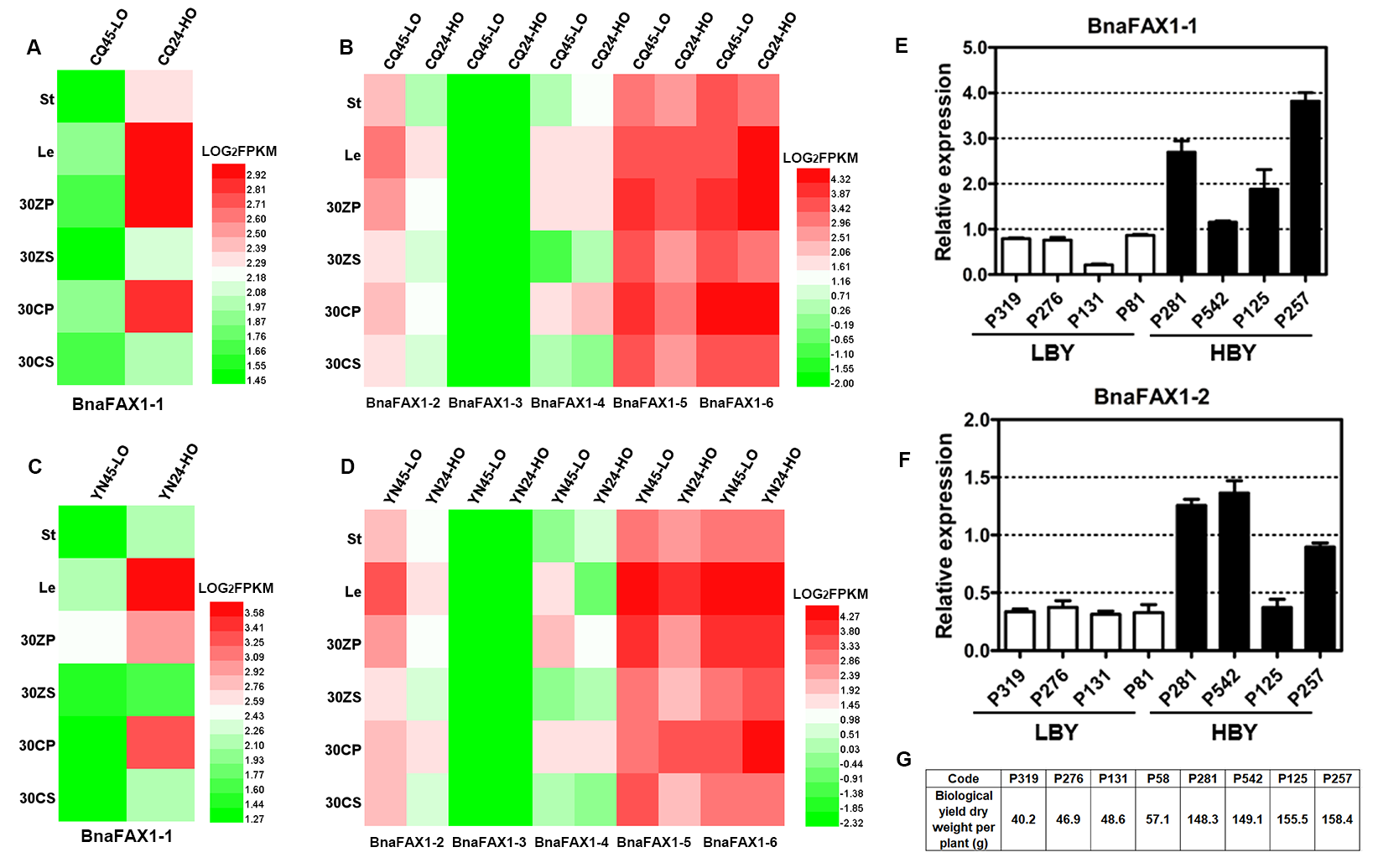


**Figure S3.** The expression levels of six members of *BnaFAX1* in six tissues of a pair of high- and low- seed oil content accessions grown in Chongqing (CQ24, CQ45) and Yunnan (YN24, YN45). CQ24, YN24 represent high- seed oil content accessions (HO); CQ45, YN45 represent low- seed oil content accessions (LO). St, Stem; Le, Leaf; 30SPM, silique pericarps on the main inflorescence of 30 days after flowering; 30SM, seeds on the main inflorescence of 30 days after flowering; 30SPB, silique pericarps on the primary branch of 30 days after flowering; 30SB, seeds on the primary branch of 30 days after flowering. The expression levels of *BnaFAX1-1* (E), *BnaFAX1-2* (F) in seedling leaves (120 days in field) of four pair with high- (P281, P542, P125, P257-HBY) and low- biological yield accessions (P319, P276, P131, P81-LBY). The biological yield dry weight per plant for each accession (G).


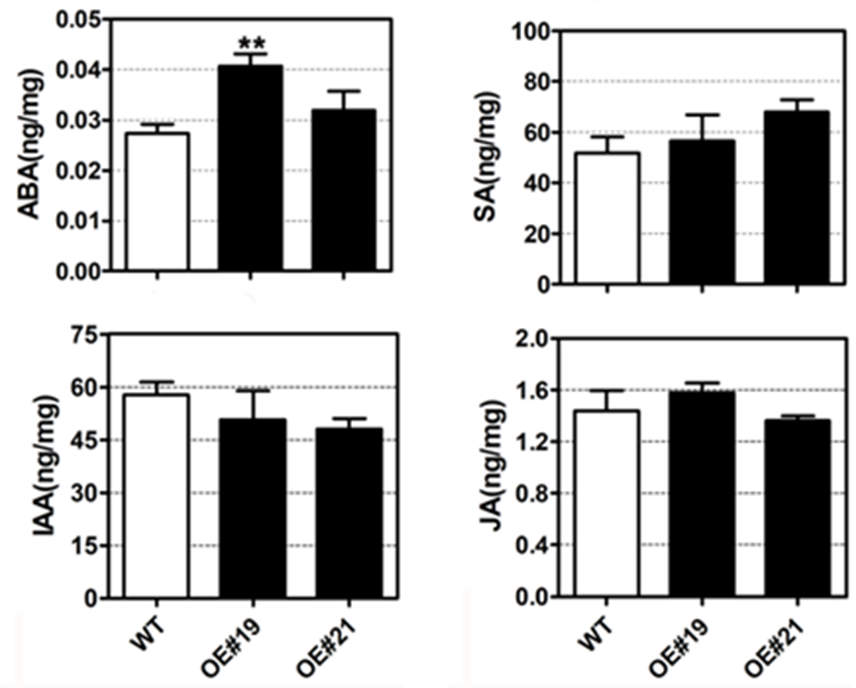


**Figure S4.** Phytohormone contents in leaves from 32-d-old WT and *B. napus* lines overexpressing *BnaFAX1-1*

ABA, abscisic acid; IAA, indole-3-acetic acid; SA, salicylic acid; JA, jasmonic acid.

**Table S1.** Summary of significant associated SNPs and candidate genes for biological yield.

| **SNPs** | **Chr.** | **Position** | **Optimal model** | **P-Value** | **R^2^** | **Confidence interval** | **Candidate genes** |
| --- | --- | --- | --- | --- | --- | --- | --- |
| Bn-A03-p15675871 | A03 | 14735140 | P+K | 1.8615E-05 | 5.40% | 14485140-14985140 | BnaA03g29780D-BnaA03g30970D |
| Bn-A03-p20375514 | A03 | 19247585 | P+K | 3.4663E-08 | 8.36% | 18997585-19497585 | BnaA03g38220D-BnaA03g39130D |
| Bn-A03-p21904842 | A03 | 20689460 | P+K | 1.1321E-05 | 5.63% | 20439460-20939460 | BnaA03g40870D-BnaA03g41700D |
| Bn-A03-p4256940 | A03 | 3794804 | P+K | 2.4131E-05 | 5.27% | 3544804-4044804 | BnaA03g07970D-BnaA03g08940D |
| Bn-A03-p7643795 | A03 | 6944642 | P+K | 2.1617E-05 | 5.33% | 6694642-7194642 | BnaA03g14570D-BnaA03g15550D |
| Bn-A03-p8475614 | A03 | 7780068 | P+K | 8.749E-06 | 5.75% | 7530068-8030068 | BnaA03g16180D-BnaA03g17110D |
| Bn-A03-p9129107 | A03 | 8414874 | P+K | 2.1261E-05 | 5.33% | 8164874-8664874 | BnaA03g17390D-BnaA03g18430D |
| Bn-A03-p18873704 | A03 | 17888586 | P+K | 1.9152E-05 | 5.38% | 17638586-18138586 | BnaA03g36090D-BnaA03g36910D |
| Bn-A03-p2948394 | A03 | 2510281 | P+K | 0.00001676 | 5.44% | 2260281-2760281 | BnaA03g04890D-BnaA03g06120D |
| Bn-A04-p12759499 | A04 | 13429641 | P+K | 1.0042E-05 | 5.68% | 13179641-13679641 | BnaA04g16080D-BnaA04g16720D |
| Bn-A04-p15108913 | A04 | 15545719 | P+K | 3.3011E-06 | 6.21% | 15295719-15795719 | BnaA04g19400D-BnaA04g20530D |
| Bn-A04-p2222852 | A04 | 1752487 | P+K | 2.3886E-05 | 5.28% | 1502487-2002487 | BnaA04g02280D-BnaA04g03030D |
| Bn-A04-p17736387 | A04 | 17846200 | P+K | 1.9021E-05 | 5.39% | 17596200-18096200 | BnaA04g23870D-BnaA04g24870D |
| Bn-A04-p8117244 | A04 | 9402482 | P+K | 1.4219E-05 | 5.52% | 9152482-9652482 | BnaA04g10420D-BnaA04g11120D |
| Bn-A05-p6542040 | A05 | 6205280 | P+K | 2.8162E-05 | 5.20% | 5955280-6455280 | BnaA05g10850D-BnaA05g11510D |
| Bn-A05-p822597 | A05 | 958179 | P+K | 9.5196E-06 | 5.71% | 708179-1208179 | BnaA05g01200D-BnaA05g02150D |
| Bn-A06-p11919396 | A06 | 14582267 | P+K | 3.3868E-06 | 6.19% | 14332267-14832267 | BnaA06g20800D-BnaA06g21360D |
| Bn-A06-p14576278 | A06 | 16129554 | P+K | 4.6608E-06 | 6.04% | 15879554-16379554 | BnaA06g22700D-BnaA06g23580D |
| Bn-A06-p16406113 | A06 | 17919297 | P+K | 6.1158E-06 | 5.92% | 17669297-18169297 | BnaA06g25470D-BnaA06g26320D |
| Bn-A06-p16994210 | A06 | 18408096 | P+K | 9.0643E-07 | 6.81% | 18158096-18658096 | BnaA06g26400D-BnaA06g27180D |
| Bn-A06-p24501540 | A06 | 23444533 | P+K | 8.3549E-07 | 6.85% | 23194533-23694533 | BnaA06g35130D-BnaA06g36090D |
| Bn-A06-p18065846 | A06 | 19443728 | P+K | 1.5754E-05 | 5.47% | 19193728-19693728 | BnaA06g27910D-BnaA06g28770D |
| Bn-A07-p12878986 | A07 | 14975988 | P+K | 1.2878E-05 | 5.57% | 14725988-15225988 | BnaA07g17690D-BnaA07g18790D |
| Bn-A07-p14594344 | A07 | 16471338 | P+K | 6.7624E-06 | 5.87% | 16221338-16721338 | BnaA07g20810D-BnaA07g21640D |
| Bn-A07-p14958606 | A07 | 16830449 | P+K | 5.4551E-06 | 5.97% | 16580449-17080449 | BnaA07g21370D-BnaA07g22300D |
| Bn-A07-p22034462 | A07 | 23418663 | P+K | 1.0932E-05 | 5.64% | 23168663-23668663 | BnaA07g33910D-BnaA07g34960D |
| Bn-scaff_15818_1-p2633791 | A07 | 15988008 | P+K | 4.2673E-06 | 6.09% | 15738008-16238008 | BnaA07g19830D-BnaA07g20830D |
| Bn-scaff_17708_1-p169681 | A07 | 1250011 | P+K | 5.0272E-07 | 7.09% | 1000011-1500011 | BnaA07g01330D-BnaA07g01810D |
| Bn-A07-p12412116 | A07 | 14546056 | P+K | 3.1127E-05 | 5.15% | 14296056-14796056 | BnaA07g16920D-BnaA07g17860D |
| Bn-A08-p1068698 | A08 | 984273 | P+K | 7.0255E-06 | 5.85% | 734273-1234273 | BnaA08g01020D-BnaA08g01470D |
| Bn-A08-p11329864 | A08 | 9184409 | P+K | 2.1932E-05 | 5.32% | 8934409-9434409 | BnaA08g09300D-BnaA08g10070D |
| Bn-A08-p12556455 | A08 | 10335312 | P+K | 2.6688E-05 | 5.23% | 10085312-10585312 | BnaA08g10940D-BnaA08g11780D |
| Bn-A08-p12752628 | A08 | 10449877 | P+K | 3.9954E-08 | 8.29% | 10199877-10699877 | BnaA08g11150D-BnaA08g11950D |
| Bn-A08-p13376746 | A08 | 11121237 | P+K | 7.8708E-06 | 5.80% | 10871237-11371237 | BnaA08g12160D-BnaA08g13020D |
| Bn-A08-p17108442 | A08 | 14587172 | P+K | 0.00002713 | 5.22% | 14337172-14837172 | BnaA08g18300D-BnaA08g19220D |
| Bn-A08-p17193797 | A08 | 14679373 | P+K | 8.6818E-07 | 6.15% | 14429373-14929373 | BnaA08g18440D-BnaA08g19360D |
| Bn-A08-p19676653 | A08 | 17007108 | P+K | 1.0642E-05 | 5.66% | 16757108-17257108 | BnaA08g23630D-BnaA08g24850D |
| Bn-A08-p20354609 | A08 | 17822431 | P+K | 9.4889E-07 | 6.79% | 17572431-18072431 | BnaA08g25590D-BnaA08g26860D |
| Bn-A08-p3444437 | A08 | 2856532 | P+K | 8.7616E-07 | 6.83% | 2606532-3106532 | BnaA08g03180D-BnaA08g03790D |
| Bn-A08-p4293226 | A08 | 3694293 | P+K | 0.00001694 | 5.44% | 3444293-3944293 | BnaA08g04110D-BnaA08g04440D |
| Bn-A08-p7814328 | A08 | 6786884 | P+K | 1.7445E-06 | 6.51% | 6536884-7036884 | BnaA08g06600D-BnaA08g06970D |
| Bn-A08-p8543675 | A08 | 7389541 | P+K | 5.9436E-07 | 7.01% | 7139541-7639541 | BnaA08g07100D-BnaA08g07720D |
| Bn-scaff_27039_1-p197834 | A08 | 7397046 | P+K | 3.8386E-06 | 6.13% | 7147046-7647046 | BnaA08g07100D-BnaA08g07730D |
| Bn-A08-p10795996 | A08 | 8702202 | P+K | 2.7903E-05 | 5.21% | 8452202-8952202 | BnaA08g08670D-BnaA08g09340D |
| Bn-A08-p1089123 | A08 | 1000449 | P+K | 2.7012E-05 | 5.22% | 750449-1250449 | BnaA08g01050D-BnaA08g01470D |
| Bn-A07-p9535820 | A09 | 19578645 | P+K | 1.6395E-05 | 5.45% | 19328645-19828645 | BnaA09g26170D-BnaA09g26610D |
| Bn-A09-p10306216 | A09 | 9527218 | P+K | 4.2695E-06 | 6.08% | 9277218-9777218 | BnaA09g15830D-BnaA09g16430D |
| Bn-A09-p33694154 | A09 | 30983144 | P+K | 1.3529E-06 | 6.62% | 30733144-31233144 | BnaA09g44840D-BnaA09g45780D |
| Bn-A09-p21224256 | A09 | 18562826 | P+K | 1.3194E-06 | 6.64% | 18312826-18812826 | BnaA09g25280D-BnaA09g25740D |
| Bn-A10-p7410271 | A10 | 9082874 | P+K | 1.4043E-05 | 5.53% | 8832874-9332874 | BnaA10g10370D-BnaA10g11050D |
| Bn-scaff_17827_1-p271972 | C01 | 7529799 | P+K | 2.6598E-06 | 6.31% | 7279799-7779799 | BnaC01g11640D-BnaC01g12310D |
| Bn-scaff_22790_1-p370880 | C01 | 30305788 | P+K | 1.1496E-05 | 5.62% | 30055788-30555788 | BnaC01g31260D-BnaC01g31670D |
| Bn-C13594816-p86 | C02 | 40111275 | P+K | 3.341E-06 | 6.20% | 39861275-40361275 | BnaC02g36850D-BnaC02g37490D |
| Bn-scaff_17623_1-p1013272 | C02 | 42483650 | P+K | 1.1354E-05 | 5.63% | 42233650-42733650 | BnaC02g39260D-BnaC02g39800D |
| Bn-A08-p293480 | C03 | 60268012 | P+K | 4.2789E-06 | 6.08% | 60018012-60518012 | BnaC03g70360D-BnaC03g71050D |
| Bn-scaff_15703_1-p53309 | C03 | 52357539 | P+K | 4.2981E-06 | 6.08% | 52107539-52607539 | BnaC03g62810D-BnaC03g63210D |
| Bn-scaff_16002_1-p1569056 | C03 | 12839150 | P+K | 4.2743E-06 | 6.08% | 12589150-13089150 | BnaC03g22760D-BnaC03g23460D |
| Bn-scaff_16182_1-p319123 | C03 | 51969206 | P+K | 8.873E-06 | 5.74% | 51719206-52219206 | BnaC03g62610D-BnaC03g62910D |
| Bn-scaff_17440_1-p761980 | C03 | 47656790 | P+K | 1.3754E-05 | 5.54% | 47406790-47906790 | BnaC03g58040D-BnaC03g58620D |
| Bn-scaff_17521_1-p807610 | C03 | 21410571 | P+K | 1.6553E-06 | 6.53% | 21160571-21660571 | BnaC03g34800D-BnaC03g35690D |
| Bn-scaff_18322_1-p918195 | C03 | 8116755 | P+K | 8.0864E-07 | 6.87% | 7866755-8366755 | BnaC03g15670D-BnaC03g16440D |
| Bn-scaff_18482_1-p437945 | C03 | 19927672 | P+K | 2.9206E-06 | 6.26% | 19677672-20177672 | BnaC03g32010D-BnaC03g32960D |
| Bn-scaff_22728_1-p360792 | C03 | 6151071 | P+K | 6.4815E-06 | 5.89% | 5901071-6401071 | BnaC03g12190D-BnaC03g13250D |
| Bn-A04-p15456188 | C04 | 44583867 | P+K | 2.9722E-05 | 4.56% | 44333867-44833867 | BnaC04g44200D-BnaC04g44970D |
| Bn-scaff_18712_1-p602624 | C04 | 14356066 | P+K | 2.6715E-06 | 6.30% | 14106066-14606066 | BnaC04g16120D-BnaC04g16530D |
| Bn-scaff_19049_1-p163830 | C04 | 27480077 | P+K | 1.1142E-06 | 6.03% | 27230077-27730077 | BnaC04g25980D-BnaC04g26330D |
| Bn-A04-p10903353 | C04 | 37955630 | P+K | 4.9185E-06 | 6.02% | 37705630-38205630 | BnaC04g36160D-BnaC04g36710D |
| Bn-scaff_16770_1-p684639 | C05 | 35242768 | P+K | 7.0506E-07 | 6.24% | 34992768-35492768 | BnaC05g35660D-BnaC05g36310D |
| Bn-scaff_18181_1-p573554 | C05 | 7093460 | P+K | 0.00001993 | 5.36% | 6843460-7343460 | BnaC05g11820D-BnaC05g12580D |
| Bn-scaff_21884_1-p235276 | C05 | 4305731 | P+K | 1.3092E-05 | 5.56% | 4055731-4555731 | BnaC05g07980D-BnaC05g08670D |
| Bn-scaff_16110_1-p1990416 | C07 | 42889038 | P+K | 8.2661E-06 | 5.78% | 42639038-43139038 | BnaC07g43730D-BnaC07g44700D |
| Bn-scaff_18181_1-p1849246 | C07 | 34322840 | P+K | 6.2863E-08 | 8.07% | 34072840-34572840 | BnaC07g29180D-BnaC07g29890D |
| Bn-scaff_22310_1-p243575 | C07 | 7893119 | P+K | 8.298E-06 | 5.77% | 7643119-8143119 | BnaC07g04880D-BnaC07g05090D |
| Bn-scaff_16197_1-p1087526 | C08 | 33007840 | P+K | 1.2155E-06 | 6.67% | 32757840-33257840 | BnaC08g34690D-BnaC08g35350D |
| Bn-scaff_16197_1-p1970599 | C08 | 32209089 | P+K | 4.226E-07 | 7.17% | 31959089-32459089 | BnaC08g33500D-BnaC08g34330D |
| Bn-scaff_16445_1-p968765 | C08 | 35812498 | P+K | 3.3183E-06 | 6.20% | 35562498-36062498 | BnaC08g40240D-BnaC08g41420D |
| Bn-scaff_16766_1-p179061 | C08 | 9447598 | P+K | 1.1372E-05 | 4.99% | 9197598-9697598 | BnaC08g06670D-BnaC08g06960D |
| Bn-scaff_18602_1-p278628 | C08 | 16438368 | P+K | 3.4965E-07 | 7.26% | 16188368-16688368 | BnaC08g10770D-BnaC08g11240D |
| Bn-scaff_21786_1-p111311 | C08 | 19631438 | P+K | 6.2857E-06 | 5.90% | 19381438-19881438 | BnaC08g14600D-BnaC08g15460D |
| Bn-scaff_23765_1-p208 | C08 | 7612606 | P+K | 1.1777E-05 | 5.61% | 7362606-7862606 | BnaC08g05480D-BnaC08g05670D |
| Bn-scaff_17088_1-p327069 | C09 | 40117533 | P+K | 1.1466E-05 | 5.62% | 39867533-40367533 | BnaC09g36540D-BnaC09g37010D |
| Bn-scaff_22082_1-p272234 | C09 | 44582838 | P+K | 4.8899E-06 | 6.02% | 44332838-44832838 | BnaC09g42950D-BnaC09g43640D |
| Bn-scaff_18100_1-p119095 | C09 | 13746357 | P+K | 0.00001696 | 5.44% | 13496357-13996357 | BnaC09g16650D-BnaC09g17210D |
| Bn-A03-p19751864 | A03 | 18716830 | P+K | 1.2742E-05 | 3.47% | 18466830-18966830 | BnaA03g37290D-BnaA03g38160D |
| Bn-A09-p35604358 | A09 | 32733944 | P+K | 2.5481E-05 | 4.38% | 32483944-32983944 | BnaA09g48510D-BnaA09g49650D |
| Bn-scaff_17109_1-p1144887 | C02 | 41209839 | P+K | 0.00002473 | 4.39% | 40959839-41459839 | BnaC02g38070D-BnaC02g38470D |
| Bn-scaff_17109_4-p95949 | C02 | 40759539 | P+K | 0.00002694 | 3.84% | 40509539-41009539 | BnaC02g37540D-BnaC02g38070D |
| Bn-scaff_18181_1-p620712 | C05 | 7041784 | P+K | 1.7199E-05 | 4.53% | 6791784-7291784 | BnaC05g11670D-BnaC05g12490D |

**Table S2. Chloroplast membrane proteins in candidate intervals associated with biological yield.**

| **Genes name** | **Homologous** | **Functional annotation** |
| --- | --- | --- |
| BnaA03g38970D | AT2G16070 | PLASTID DIVISION2 (PDV2) |
| BnaA03g05210D | AT3G46740 | translocon at the outer envelope membrane of chloroplasts 75-III (TOC75-III) |
| BnaC09g42950D | AT5G13720 | Uncharacterised protein family (UPF0114) |
| BnaC09g43360D | AT5G13720 | Uncharacterised protein family (UPF0114) |
| BnaA07g34270D | AT1G78620 | Protein of unknown function DUF92, transmembrane |
| BnaC07g04930D | AT2G15290 | translocon at inner membrane of chloroplasts 21 (TIC21) |
| BnaA03g08670D | AT5G22790 | reticulata-related 1 (RER1) |
| BnaA04g02480D | AT3G57280 | FAX1, a Novel Membrane Protein Mediating Plastid Fatty Acid Export |
| BnaA07g17240D | AT3G57280 | FAX1, a Novel Membrane Protein Mediating Plastid Fatty Acid Export |
| BnaC07g29780D | AT5G24690 | INVOLVED IN: biological_process unknown |
| BnaC03g32140D | AT4G00370 | ANTR2 |
| BnaA03g41190D | AT3G51140 | Protein of unknown function (DUF3353) |
| BnaA05g10910D | AT2G32480 | putative (SITE-2/S2P)-like metalloprotease (AtS2P1) |
| BnaC07g43940D | AT4G32770 | VITAMIN E DEFICIENT 1 (VTE1) |
| BnaA08g07530D | AT4G31780 | monogalactosyl diacylglycerol synthase 1 (MGD1) |
| BnaA04g02830D | AT3G56940 | COPPER RESPONSE DEFECT 1 (CRD1) |
| BnaA07g17040D | AT3G56940 | COPPER RESPONSE DEFECT 1 (CRD1) |
| BnaA08g18750D | AT1G27770 | autoinhibited Ca2+-ATPase 1 (ACA1) |
| BnaC03g16230D | AT5G50920 | CLPC homologue 1 (CLPC1) |
| BnaC03g58140D | AT1G27770 | autoinhibited Ca2+-ATPase 1 (ACA1) |
| BnaA08g19310D | AT1G25290 | RHOMBOID-like protein 10 (RBL10) |
| BnaA09g48840D | AT1G08570 | atypical CYS HIS rich thioredoxin 4 (ACHT4) |
| BnaA03g15390D | AT2G33380 | RESPONSIVE TO DESSICATION 20 (RD20) |
| BnaA04g19410D | AT2G33380 | RESPONSIVE TO DESSICATION 20 (RD20) |
| BnaC03g16180D | AT5G51020 | plastidial protein of unknown function, affects pattern of plastid/cell division (AtCLR) |
| BnaA03g05720D | AT3G16620 | translocon outer complex protein 120 (TOC120) |
| BnaA03g08310D | AT5G21990 | Tetratricopeptide repeat (TPR)-like superfamily protein (TPR7) |
| BnaA06g27960D | AT5G25900 | Protein of unknown function |
| BnaC09g36600D | AT5G21990 | Tetratricopeptide repeat (TPR)-like superfamily protein (TPR7) |

**Table S3. Identification of BnaFAX gene family members**

| **Isoforms** | **Transcript name** | **At Orthologs** | **Location** | **gDNA size (bp)** | **Exon** | **CDS size (nts)** | **Peptide residues** | **Theoretical pI** | **Theoretical Mw (Da)** | **Number of Transmembrane** |
| --- | --- | --- | --- | --- | --- | --- | --- | --- | --- | --- |
| BnaFAX1-1 | BnaA07g17240D | AT3G57280 | 14454789-14456198 | 1410 | 6 | 681 | 226 | 9.75 | 24599.52 | 3 |
| BnaFAX1-2 | BnaCnng07490D | AT3G57280 | 6819666-6821289 | 1624 | 6 | 687 | 228 | 9.65 | 24937.78 | 3 |
| BnaFAX1-3 | BnaA04g02480D | AT3G57280 | 1630801-1631454 | 654 | 4 | 372 | 123 | 9.79 | 13017.62 | 2 |
| BnaFAX1-4 | BnaC04g24320D | AT3G57280 | 25311625-25312966 | 1342 | 6 | 528 | 175 | 9.94 | 19209.43 | 3 |
| BnaFAX1-5 | BnaC08g28200D | AT3G57280 | 28954480-28956120 | 1641 | 7 | 669 | 222 | 9.76 | 23900.51 | 3 |
| BnaFAX1-6 | BnaA09g36620D | AT3G57280 | 26442165-26443778 | 1614 | 7 | 657 | 218 | 9.79 | 23577.16 | 3 |
| BnaFAX2-1 | BnaC04g06730D | AT2G38550 | 4841234-4843565 | 2332 | 6 | 1011 | 336 | 5.97 | 37058.99 | 3 |
| BnaFAX2-2 | BnaA05g34770D | AT2G38550 | 326592-329048 | 2457 | 6 | 1011 | 336 | 6.43 | 37006.08 | 3 |
| BnaFAX3-1 | BnaCnng12010D | AT3G43520 | 11443962-11445322 | 1361 | 3 | 705 | 234 | 9.3 | 23808.5 | 4 |
| BnaFAX3-2 | BnaA06g19610D | AT3G43520 | 12114425-12115576 | 1152 | 3 | 660 | 219 | 9.07 | 22359.84 | 4 |
| BnaFAX4-1 | BnaCnng30070D | AT1G33265 | 28523425-28524983 | 1559 | 4 | 528 | 175 | 10.02 | 18241.46 | 4 |
| BnaFAX4-2 | BnaA09g23790D | AT1G33265 | 16423783-16425150 | 1368 | 4 | 528 | 175 | 9.96 | 18151.37 | 4 |
| BnaFAX5-1 | BnaC06g41000D | AT3G20510 | 378280-379102 | 823 | 3 | 372 | 123 | 9.87 | 13091.8 | 3 |
| BnaFAX5-2 | BnaC06g04100D | AT3G20510 | 4788144-4789076 | 933 | 3 | 372 | 123 | 9.87 | 13083.73 | 3 |
| BnaFAX6-1 | BnaAnng37060D | AT3G20510 | 42019980-42021500 | 1521 | 3 | 360 | 119 | 9.7 | 12552.1 | 4 |
| BnaFAX6-2 | BnaCnng46150D | AT3G20510 | 45462307-45463756 | 1450 | 3 | 360 | 119 | 9.62 | 12535.13 | 4 |
| BnaFAX6-3 | BnaC03g41620D | AT3G20510 | 26546686-26551669 | 4984 | 13 | 2550 | 849 | 8.38 | 94334.62 | 4 |
| BnaFAX6-4 | BnaA05g20430D | AT3G20510 | 15796433-15797561 | 1129 | 3 | 360 | 119 | 9.81 | 12439.98 | 4 |
| BnaFAX6-5 | BnaA03g35780D | AT3G20510 | 17481243-17483789 | 2547 | 7 | 828 | 275 | 9.26 | 30066.92 | 4 |
| BnaFAX7-1 | BnaC08g33110D | AT2G26240 | 31734479-31736535 | 2057 | 5 | 441 | 146 | 10.15 | 15697.79 | 3 |
| BnaFAX7-2 | BnaA09g40650D | AT2G26240 | 28550586-28552778 | 2193 | 5 | 408 | 135 | 9.76 | 14336.94 | 3 |

**Table S4. A List of primer sequences used in the present study.**

| **Oligo Name** | **Sequence(5'to 3')** | **Length(bp)** |
| --- | --- | --- |
| BnaFAX1-1 FP(XbaI) | TCTAGAATGGCGACGAAAATCTCTCACC | 28 |
| BnaFAX1-1 RP(SacI) | GAGCTCTCAGTGTGAAGGGCTAGTAGATGG | 30 |
| AtFAX1 FP(SpeI) | ACTAGTATGGCTTCACAAATCTCTCAGC | 28 |
| AtFAX1 RP(BamHI) | GGATCCTCAGTATGAAGGACTAGTCGCAGATGG | 33 |
| BnaFAX1-1 FP(SpeI) | ACTAGTATGGCGACGAAAATCTCTCA | 26 |
| BnaFAX1-1 RP(BamHI) | GGATCCGTGTGAAGGGCTAGTAGATGGT | 28 |
| F35S3ND | GGAAGTTCATTTCATTTGGAGAG | 23 |
| Nos5ND | TGCCAAATGTTTGAACGATCGGG | 23 |
| qRT-BnaFAX1-1 FP | GATGGGAACAGCTCAGAAACAC | 22 |
| qRT-BnaFAX1-1 RP | GTTCCTCTACTGTCTCAGTGA | 21 |
| BnActin7-FP | TGGGTTTGCTGGTGACGAT | 19 |
| BnActin7-RP | TGCCTAGGACGACCAACAATACT | 23 |
| AtActin2-FP | GGTAACATTGTGCTCAGTGGTGG | 23 |
| AtActin2-RP | AACGACCTTAATCTTCATGCTGC | 23 |
| qRT CPS-FP | CCAGTGTCTTTCCTGTGGATC | 21 |
| qRT CPS-RP | CAATGTCTTGGACATGGGAAC | 21 |
| qRT KAO1-FP | CTCTTACGGCCTTCCGAGAAG | 21 |
| qRT KAO1-RP | CCTTCCCATCTTGAAGGATCA | 21 |
| qRT KAO2-FP | GGAACTCATTGGCAAGAAGTC | 21 |
| qRT KAO2-RP | CATGAGTCAAGAACTCGATTTC | 22 |
| qRT GA20OX1-FP | GTGACGACAAGAGCCGCCCAA | 21 |
| qRT GA20OX1-RP | AATGATTTGAAGTAGTCTCCGG | 22 |
| qRT GA20OX3-FP | GATGCCATGAACATTCTCTC | 20 |
| qRT GA20OX3-RP | TCCGGTTGGTTGCACTGTGG | 20 |
